# Novel Ambient Oxidation Trends in Fingerprint Aging Discovered by Kendrick Mass Defect Analysis

**DOI:** 10.1021/acscentsci.2c00408

**Published:** 2022-09-21

**Authors:** Andrew E. Paulson, Young Jin Lee

**Affiliations:** Department of Chemistry, Iowa State University, Ames, Iowa 50011, United States

## Abstract

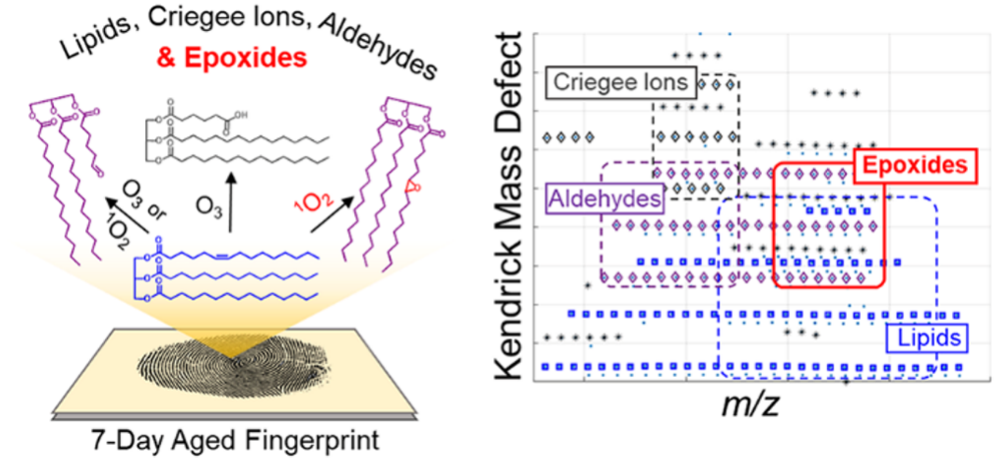

A Kendrick mass defect (KMD) plot is an efficient way to disperse complex high-resolution mass spectral data in a visually informative two-dimensional format which allows for the rapid assignment of compound classes that differ by heteroatom content and/or unsaturation. Fingerprint lipid oxidation has the potential to be used to estimate the time since deposition of a fingerprint, but the mass spectra become extremely complex as the lipids degrade. We apply KMD plot analysis for the first time to sebaceous fingerprints aged for 0–7 days to characterize lipid degradation processes analyzed by MALDI-MS. In addition to the ambient ozonolysis of fingerprint lipids previously reported, we observed unique spectral features associated with epoxides and medium chain fatty acid degradation products that are correlated with fingerprint age. We propose an ambient epoxidation mechanism via a peroxyl radical intermediate and the prevalence of omega-10 fatty acyl chains in fingerprint lipids to explain the features observed by the KMD plot analysis. Our hypotheses are supported by an aging experiment performed in a sparse ozone condition and on-surface Paternò–Büchi reaction. A comprehensive understanding of fingerprint degradation processes, afforded by the KMD plots, provides crucial insights for considering which ions to monitor and which to avoid, when creating a robust model for time since deposition of fingerprints.

## Abstract 2

A deeper understanding of fingerprint aging is gained through the Kendrick mass defect plot-guided discovery of new mass spectral trends, providing a foundation for fingerprint age estimations.

## Introduction

Fingerprints have been a mainstay in forensic investigations since the early 1900s, due to the evidentiary value of their morphology.^1^ Recently, mass spectrometry has been explored to extend the evidentiary value within a fingerprint. These efforts include but are not limited to analyzing exogenous and/or endogenous compounds within the fingerprint for suspect specific chemical information^2−7^ and assessing the diffusion of compounds within fingerprints^8,9^ or monitoring degradation of fingerprint compounds^10−12^ for time since deposition estimations. Time since deposition, or fingerprint aging, is of special interest because such temporal evidence describes the relevance of a fingerprint to the timeline of a crime. Despite many attempts to address this gap in fingerprint forensic evidence, reliable time since deposition estimations have remained a challenge due to many reasons such as environmental considerations, fingerprint composition and formation, and surface characteristics.^13^

Recently, we proposed that the ambient ozonolysis that occurs in unsaturated triacylglycerols (TGs) of sebaceous fingerprints has the potential to be used to estimate the time since fingerprint deposition within a week.^10^ The overall aging process, however, is much more complex because the ozonolysis oxidation products are intermediates and can further degrade when they contain multiple degrees of unsaturation. Additionally, other endogenous unsaturated lipids such as squalene (SQ), wax esters (WEs), fatty acids (FAs), and diacylglycerols (DGs), likely compete for the ambient ozone during degradation.^10^ Consequently, though the initial composition of sebaceous fingerprints is relatively simple, comprised mostly of TGs, SQ, WEs, FAs, and DGs, the aging process produces a complex mixture of many degradation products. High-resolution mass spectrometry (HRMS) can untangle some of this complexity; however, the data analysis is still a daunting task without an efficient strategy.

Kendrick mass defect (KMD) analysis allows for the rapid grouping of congener ions into compound classes that have elemental compositions with the same heteroatom (O, N, P, S) content and unsaturation, a homologous series. The Kendrick mass (KM) was proposed in 1963 as a way to simplify spectral interpretation by normalizing the *m*/*z* values to 14 being the mass of the ^12^C^1^H_2_, alkyl chain unit.^14^
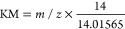


Compounds of similar composition, differing only in the number of CH_2_ units, have the same KMD, the difference between the rounded KM and KM, but differing KM. Further, compounds with the same heteroatom class can be easily identified in a two-dimensional KMD plot of the mass-to-charge ratio (*m*/*z*) vs the KMD. In the KMD plots, homologous series align horizontally, and parallel homologous series with a ΔKMD of ±0.0134 differ by a degree of unsaturation. The KMD plot has been extensively utilized in HRMS analysis of petroleum^15^ but has also been extended to biofuel,^16^ polymer,^17^ environmental research,^18^ as well as lipidomics.^19,20^ In forensics, it has only been applied for synthetic designer drugs in order to streamline drug analog identification.^21^

Here we propose the use of KMD plots as a visual tool to streamline the identification of compound classes associated with fingerprint aging. Building upon our previous findings relating to ambient ozonolysis,^10^ KMD analysis comprehensively reveals the molecular details of fingerprint aging processes. Our analysis led to the discovery of a new series of epoxidation products, attributed to epoxidation of carbon–carbon double bonds in TGs, WEs, FAs, and DGs by singlet oxygen. Our KMD analysis also led us to find a new aging trend associated with medium chain fatty acids (MCFA), especially FA 10:0, and other considerations for developing time since deposition models.

## Results and Discussion

### Subtracted Spectra and KMD Plots for General Spectral Differences

**Figure 1:**
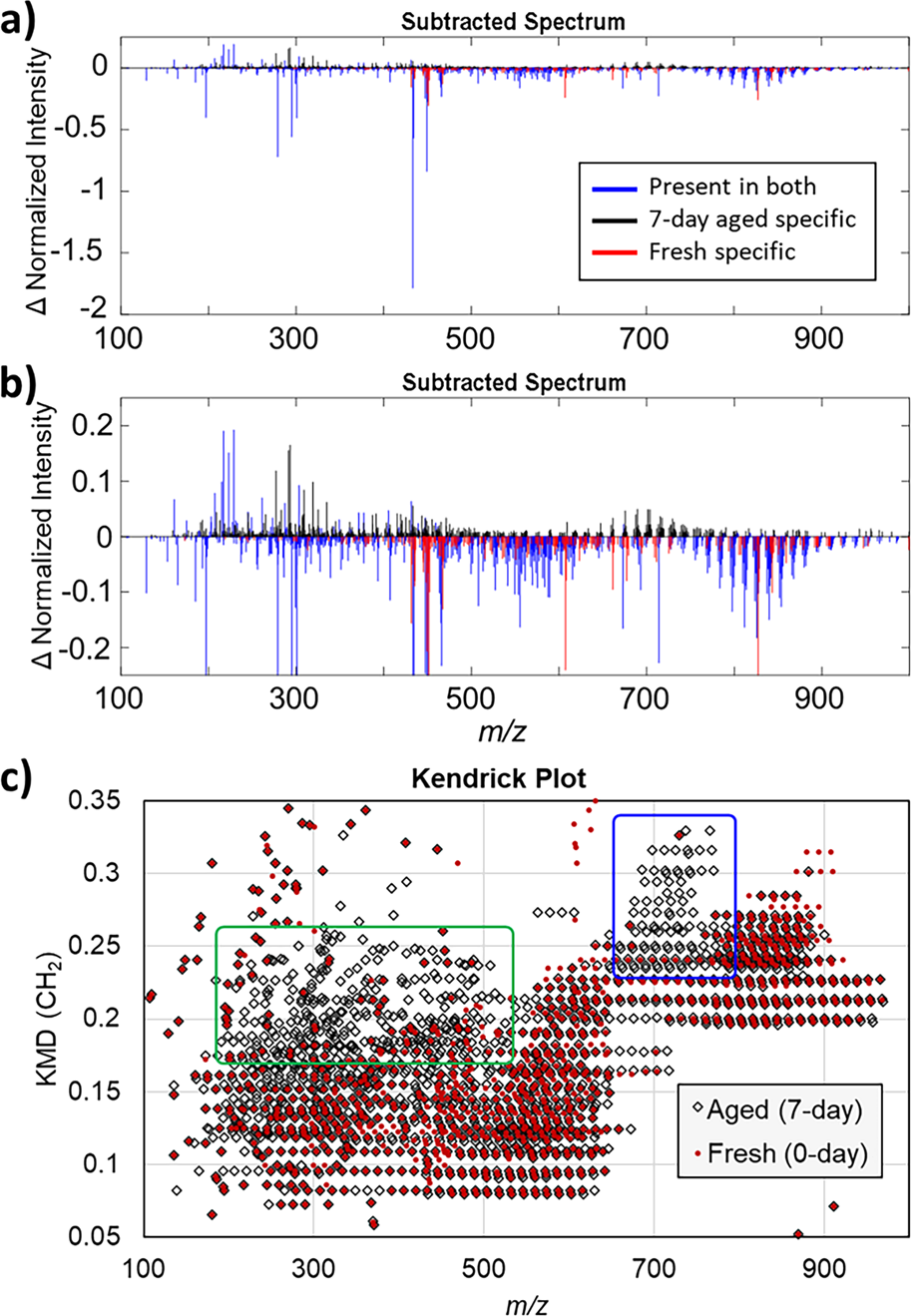
(a) Fresh fingerprint MALDI-MS spectrum subtracted from a 7-day fingerprint spectrum. (b) Zoomed-in subtracted spectrum in the *y*-axis. (c) Overlain KMD plot of the fresh and 7-day-old fingerprints.

A high-resolution Orbitrap MS connected to a MALDI source is used in this study for the analysis of aged fingerprints in ambient lab conditions. Figure 1 is the subtracted spectrum and the overlain KMD plots in the lipid KMD range (0.05–0.35) for the fresh (0-day) and 7-day aged fingerprints. To generate the subtracted spectrum, all spectral features were normalized to the summed signal of saturated TGs to account for differences in deposition. The original spectra (Figure S1), full overlain KMD plot (Figure S2), and environmental conditions during the aging (Figure S3, Table S1) can be found in the Supporting Information. KMD bubble plots are also helpful as they retain the relative abundance information of the plot features (Figure S4).

Many noticeable differences can be found in the subtracted mass spectra. Most notable is the loss of SQ (*m*/*z* 433.3805) in the aged fingerprint (Figure 1a). This is consistent with previous findings of rapid decay of SQ in aged fingerprints analyzed by GC-MS and LC-MS.^22,23^ Other differences, such as the negative peaks in *m*/*z* 700–900 and positive peaks in *m*/*z* 600–800 (Figure 1b), are related to the fingerprint aging and are clearly distinguished in the KMD plot (Figure 1c). Thus, the spectral and plot differences can be used in concert in order to identify time-dependent *m*/*z* features and related series to understand the molecular details of the aging process over time. Previously, we proposed to use the degradation of TGs to monitor the time since deposition of fingerprints due to high ion abundance and multiple levels of unsaturation.^10^ The ozonolysis products for TGs are readily observed as the new cluster of KMD plot features in the *m*/*z* range of ∼675–775 (blue box). However, oxidative aging of WEs, DGs, FAs, and SQ (green box) can also be studied using MALDI-HRMS with KMD analysis as described in more detail later.

### Generating *in Silico* Theoretical Heteroatom Class List

**Scheme 1:**
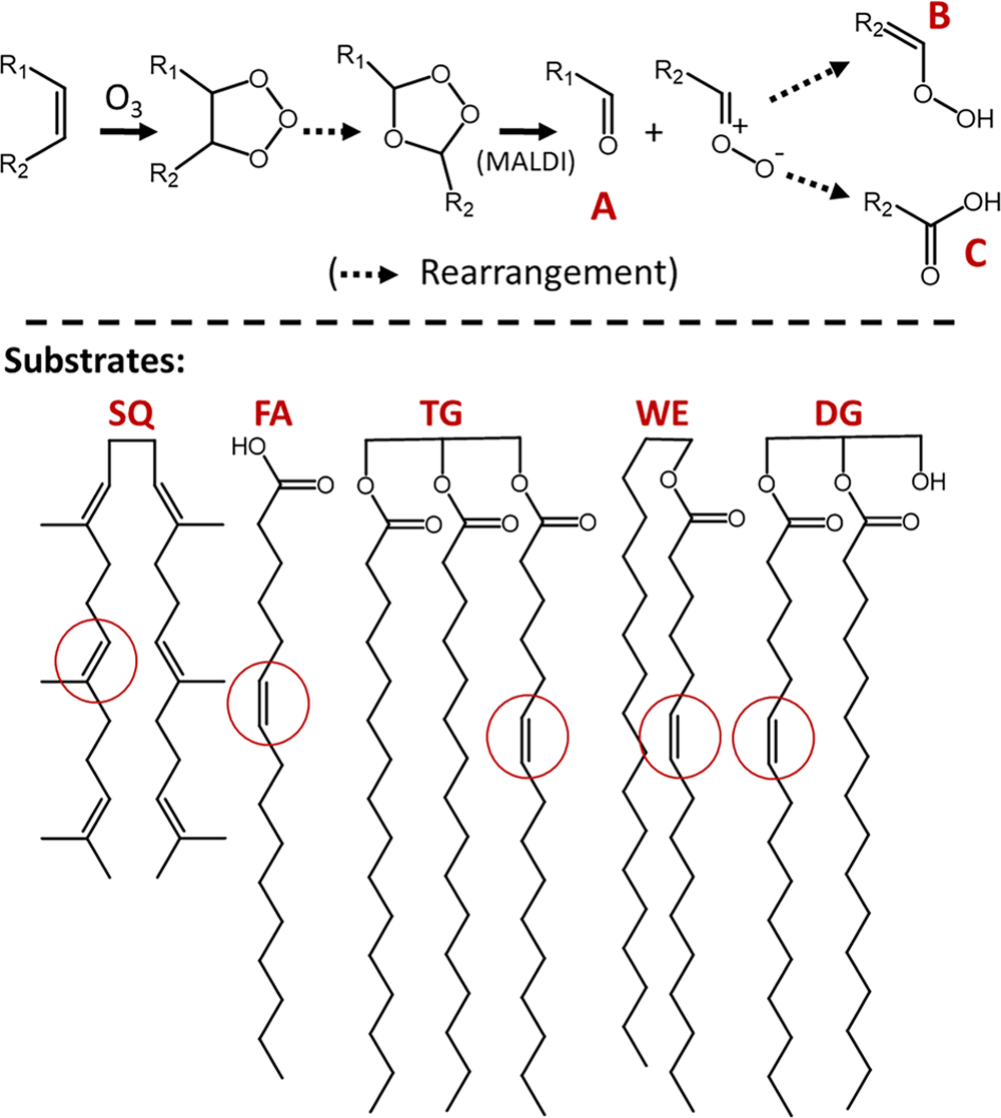
Ambient Ozonolysis of Unsaturated Lipids in Fingerprints

Given the ease of visualizing the differences in plot features between the aged and fresh prints by using the subtracted spectrum and KMD plot, the subsequent task is assigning elemental compositions. Considering that TGs, WEs, DGs, FAs, and SQ can be readily detected in our MALDI condition and may undergo ambient ozonolysis,^10,24^ we made an *in silico* theoretical target list for each elemental composition that would arise from the ozonolysis process as shown in Scheme 1. SQ was omitted from heteroatom class consideration as it is not expected to have a homologous series and is anticipated to rapidly oxidize into volatile compounds. Ambient ozone molecules react with any carbon–carbon double bonds in lipid substrates to form primary ozonides, which then rearrange to secondary ozonides. The secondary ozonide is relatively stable but breaks down slowly into the aldehyde (A) even in the ambient environment or into the aldehyde (A) and Criegee ion isomers (B or C) by in-source fragmentation during MALDI analysis.

Most substrates in Scheme 1 can have multiple carbon-carbon double bonds which may lead to sequential ozonolysis. Therefore, a second ozonolysis process was also included for DGs, WEs, and TGs in the KM target list. The target list of heteroatom classes was created to cover the entire spectral range in order to assess any unexpected or conflicting plot features that reside in the same heteroatom class. Given that sodium acetate was used as an additive to promote cationization in positive ion mode, sodium ion adducts were the primary adducts used to produce the target list. Carboxylic acids (FA or product **C** in Scheme 1) are detected as disodiated, sodium adducts of sodium carboxylate, and these theoretical mass values were also included. Lastly, all classes were allowed one sodium to be replaced with potassium, in order to understand the contribution of potassium adducts to the spectral complexity; however, their contribution has a minimal impact on the interpretation based on sodium adducts. The theoretical heteroatom classes have the general form of C_c_H_2c-Z_O_w_Na_*x*_K_*y*_. Z-values in the formulas are related to double bond equivalents. Table S2 summarized the searched heteroatom classes, double bond equivalent (DBE) values, minimum Z-values, and their molecular constituents.

### KMD Plot Annotations of Heteroatom Classes in Fingerprints

On the basis of the *in silico* heteroatom class list, most of the plot features in the lipid region of fresh and aged fingerprints are annotated (±2 ppm) into one of the heteroatom classes in the KMD plots (Figure S5). The mass resolving power used is sufficient to resolve Type-II isotopic overlap (Δ*m*/*z* of 0.0089 between the second ^13^C peak and a lipid with one more saturation).^25^ As we are approaching the limits of the resolving power, some ^13^C peaks are unresolved when the relative ion signal is very low (Figure S6). Regardless, it has minimal impact on the heteroatom class assignment based on the ±2 ppm mass tolerance.

**Figure 2:**
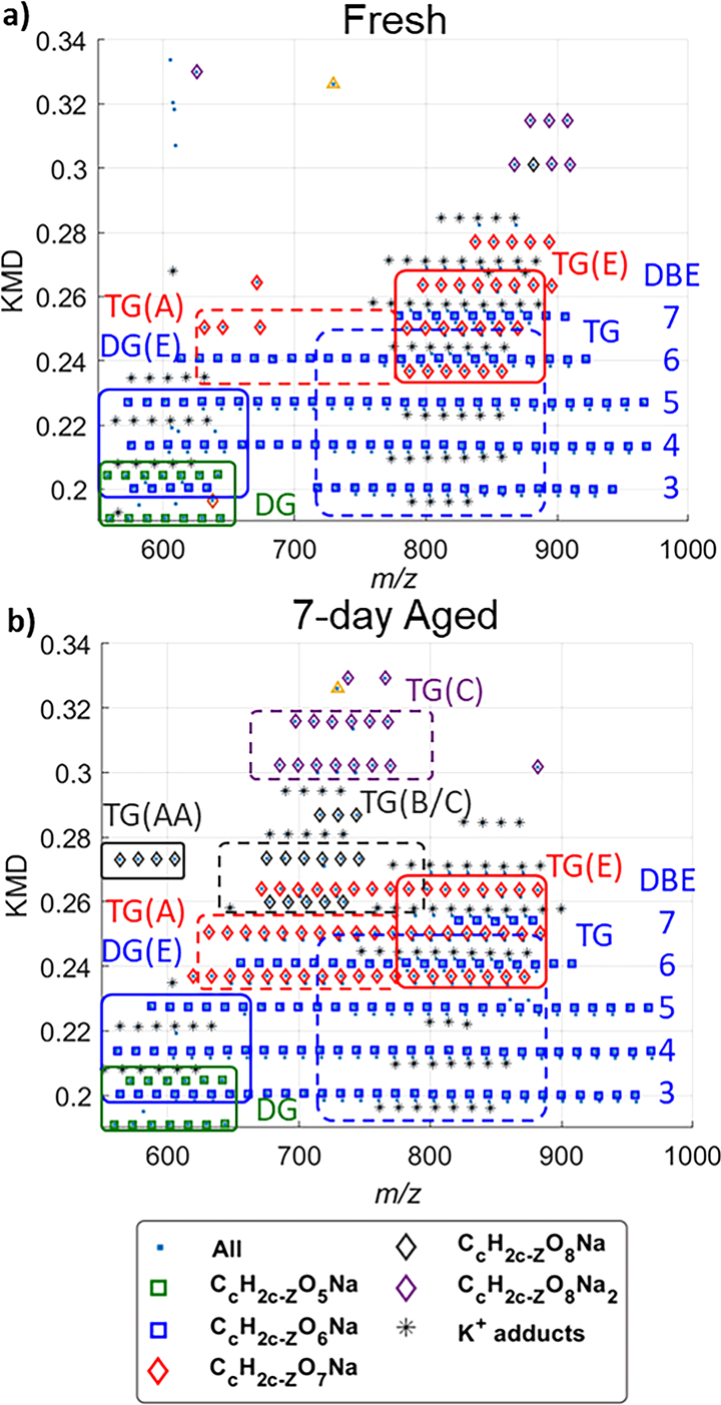
KMD plots for the (a) fresh and (b) 7-day-old fingerprints of the TG region with *S*/*N* > 30. Heteroatom class annotations are based solely on theoretical values in ambient ozonolysis (Scheme 1). Note the DBE of saturated TGs is three due to the three ester groups.

A smaller region of the annotated KMD plot is captured in Figure 2 for the *m*/*z* of 550–1000, focusing on TGs. Substrate abbreviations followed by a letter in parentheses indicate the ozonolysis product from Scheme 1. For example, TG(A) and TG(C) indicate the **A** and **C** product of TG, respectively. TG(B/C) indicates the chemical series that are associated with the isomeric single sodiated Criegee ions, product **B** or **C** in Scheme 1. Two ozonolysis processes are also possible as observed by TG(AA), aldehyde products in two fatty acyl chains of TG, but generally have low signals. Further, in Figures 2 and S5, squares (□) represent heteroatom classes mostly dominated by unreacted lipids, while diamonds (◊) and triangles (Δ) indicate series where the predominant signals are expected to be from one or two ozonolysis processes, respectively. Potassium adducts for all heteroatom classes are also included and are denoted as black asterisks (*). The color of the symbols is chosen to make a heteroatom class stick out among its neighboring heteroatom classes. A heteroatom class is comprised of homologous series aligned horizontally at the same KMD. Parallel homologous series with a KMD difference of 0.0134 indicates a difference in unsaturation; DBEs are shown for TGs as an example in Figure 2, and saturated TGs have a DBE of 3 from three ester groups.

Because the *in silico* list, based on ozonolysis products of all lipid substrates, defines most of the plot features in the aged and fresh fingerprints in Figure 2 for TG and in Figure S5 for all lipids, we are confident ambient ozonolysis plays a central role in fingerprint aging. In general, the lower mass region of the plot gets more populated (*m*/*z* < 500), consistent with oxidative degradation products. Though two sequential ozonolysis processes on the same substrate are possible for a polyunsaturated lipid, the overall contribution is minimal after 7 days. Most of the searched but absent heteroatom classes are associated with double ozonolysis products, labeled as “x” in Table S2. Investigating unannotated features is aided with using different KMD plot normalization techniques which are demonstrated and described in the Supporting Information (Supporting discussion, Figures S7–S9).

### Epoxidation: A Spectral Trend Observed by 2D KMD Plots

**Figure 3:**
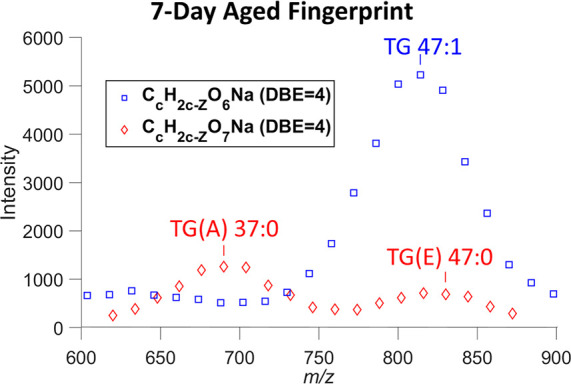
Intensity profile of C_c_H_2c-Z_O_6_Na (DBE = 4) and C_c_H_2c-Z_O_7_Na (DBE = 4) homologous series in a 7-day aged fingerprint.

Most of the aged products could be explained with ambient ozonolysis in Scheme 1 and are labeled as “TG(A)”, “TG(C)”, and “TG(B/C)” in Figure 2. Some aldehyde products, consistent with ozonolysis, are present even in fresh fingerprints, but they are in very low abundance and attributed to oxidation on the skin surface before the fingerprint is deposited. However, there are some trends that cannot be explained by ambient ozonolysis. Specifically, the cluster of plot features labeled as TG(E) with a solid red box cannot be explained as any of the substrates or products in Scheme 1. They have the same heteroatom class with TG(A) boxed with a dashed line, but their masses are in the range of the most abundant TGs, 770–900. This point is clearly demonstrated in the intensity profile along the same KMD. As shown in Figure 3, C_c_H_2c-Z_O_7_Na (DBE = 4) heteroatom class (red) has a bimodal distribution. The distribution centered at *m*/*z* ≈ 690 is TG(A) after losing an alkyl chain below the reacted double bond site from the TG ozonide. However, the distribution centered at *m*/*z* ≈ 825 has a profile similar to its potential precursor, monounsaturated TG (C_c_H_2c-Z_O_7_Na with DBE = 4, blue), and cannot be explained as TG(A). These features are also localized to the fingerprint, as seen in the MS images (Figure S10).

**Scheme 2:**
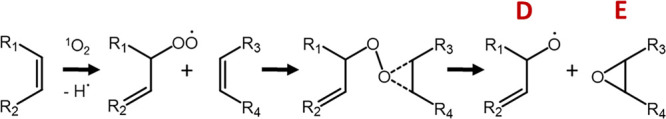
Proposed Ambient Epoxidation Mechanism, Where the Initial Hydrogen Abstraction Is at an Allylic Carbon at R_2_

To address this trend, unexplainable by ozonolysis, we hypothesize that this series is associated with an epoxidation process induced by ambient singlet oxygen (Scheme 2). Zhou et al. proposed an ambient singlet oxygen mechanism to explain the aldehyde products in ambient surface oxidation of unsaturated lipids,^26^ but it does not involve epoxidation. Weiny et al. studied the autoxidation of polyunsaturated FAs (PUFA) on monolayer thin films at 37 °C, detecting epoxide formation and confirming its structure with NMR. The mechanism proposed by Weiny et al. involves a peroxyl radical intermediate, the same as Zhou et al.’s, but the epoxides are suggested as the final products.^26,27^ Therefore, singlet oxygen is a promising source for peroxyl radical intermediates in ambient conditions to explain the subsequent epoxide formation, though other autoxidation mechanisms may also lead to the peroxyl radical. There is contradictory literature evidence for the initial hydrogen abstraction. Specifically, Wu et al. suggested that minimal epoxidation occurs for lipid monolayers or bulk systems containing exclusively monounsaturated aliphatic chains under heat.^28^ Thus, further investigation is necessary for the origin of peroxyl radical intermediate formation in fingerprint lipids.

SQ epoxides have also been observed in aged fingerprints,^23,29^ but the epoxides of TGs, WEs, FAs, and DGs have not previously been reported in fingerprints. However, epoxides of many of these lipid species have been monitored in other systems.^27,30−32^ We extend Weiny’s mechanism to include all unsaturated lipids and propose that the peroxyl radical is also generated by singlet oxygen in ambient condition as simplified in Scheme 2. This reaction scheme is consistent with the *m*/*z* values of TG(E) for having one more oxygen without alkyl chain loss from TG. The same trend is also found for other lipids as shown in Figure S5 as DG(E), WE(E), and FA(E). The epoxides are present even in the fresh sample, probably occurring on the skin surface before deposited, and carry over into the aged samples similar to TG(A). ESI-MS/MS of TG(E) from the fingerprint extracts was not successful due to poor signals, but MS/MS of FA(E) 16:0 from the same extract suggests the presence of epoxides (Figure S11). Additionally, ESI-MS/MS on the extract of aged TG standards, a mixture of TG 48:0 and TG 50:1, have fragmentation patterns consistent with epoxides (Figure S12). However, we cannot avoid the possibility that some isomeric species may also contribute to the features assigned as epoxides, such as the termination of structure D (Scheme 2) with another abstracted hydrogen to form a hydroxyl group.

To support the ambient epoxidation hypothesis, we performed an experiment in a sealed climate chamber with minimal ozone and similar or elevated levels of singlet oxygen. The relative humidity and temperature were maintained at levels similar to the ambient condition, but ozone concentration was about five times less. UVA is provided to produce singlet oxygen species from ambient oxygen;^33,34^ however, the amount of singlet oxygen was not monitored. After 3 days of aging in the sparse ozone (2.7 ± 0.7 ppb) environment, the TG(E) signal is significantly greater than that of the TG(C) ozonolysis product (*p* < 0.001; Figure S13a), in contrast to the ambient environment where it is comparable or lower (Figure S13b). The preferential TG epoxidation, as opposed to TG ozonolysis, is consistent with our singlet oxygen hypothesis. Singlet oxygen has been suggested as an oxidant in the ambient environment^26^ and has multiple routes of formation, some including origins from ozone.^35,36^

We also ruled out the possibility of other epoxide reactions. One potential explanation is peroxide containing facial cleansers, but the individual does not use such products. Additionally, TG(E) is observed in aged unsaturated TG standards as well as the fingerprints of other people. Another possibility is that epoxide formation occurs as a sample preparation artifact during the Au sputtering process (e.g., discharge excitation of oxygen impurity), but the epoxide spectral features were still present when using an organic MALDI matrix (Figure S14). Lastly, artificial epoxide formation may also happen in the laser plume plasma environment, if oxygen impurity in the MALDI source is excited by the laser. To address this possibility, nitrogen gas (99.995+%) was used to displace any oxygen that might be present in the 7.5 Torr MALDI source during analysis, but the spectral features remained the same (Figure S14).

It is important to distinguish the epoxide features from other features. For example, DG(E) has the same heteroatom class as TG and DG(A) for having O_6_, and TG(E) has the same heteroatom class as TG(A) for having O_7_, but they are differentiated by a significant mass difference. With aging, the heteroatom class broadens out significantly as low abundance lipids get oxidized, eventually overlapping, as can be seen for TG(E) and TG(A) in Figure 2b. Despite the overlap in the KMD plot, they are well separated from each other in the intensity profile as demonstrated in Figure 3 for TG(E) vs TG(A). Another example is shown in Figure S15 for DG(E), DG(A), and TG.

**Figure 4:**
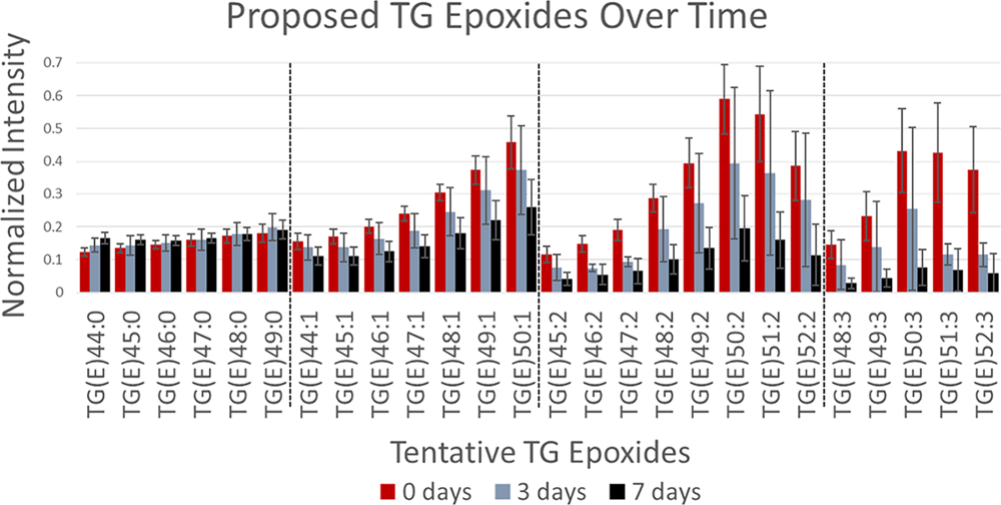
Monitoring TG(E) species during fingerprint aging. Epoxides are normalized to the corresponding saturated TG (e.g., TG(E)44:1/TG44:0). Error bars represent one standard deviation from four replicates.

To evaluate the use of the epoxides for time-since-deposition estimations, the intensities of TG(E) species were monitored in fingerprints aged for different times, as summarized and grouped into the same degree of unsaturation in Figure 4. An interesting trend is observed depending on the unsaturation of the epoxides. The epoxides of monounsaturated TGs no longer have reactive double bonds (i.e., TG X:1 becomes TG(E) X:0) and appear to remain stagnant through the aging process. The stability of the homologous series suggests that the carbon–carbon double bond is likely no longer present, consistent with fully saturated epoxides instead of a hydroxyl group. In contrast, the unsaturated epoxide species decrease in intensity over time, especially those that are multiply unsaturated. As reported by Wu et al. on the autoxidation of unsaturated FAs to epoxides, we hypothesize ambient epoxidation occurs rather rapidly but almost exclusively on the top monolayer.^28^ The epoxides would then stay at the same concentration if there is no additional unsaturation or degrade over time through the ozonolysis of other double bonds. Weiny et al. used the monolayers of PUFA adsorbed on silica gel particles to get a high yield of the epoxides^27^, following Wu et al.^28^ As ambient epoxidation occurs mostly on the top monolayers, its impact is minimal as a competing process for ozonolysis substrates and confined only to very early fingerprint aging. It is possible that multiply unsaturated epoxides might be used to model time since deposition given that they further oxidize by ozonolysis.

### Medium Chain Fatty Acid Ozonolysis Product: A Spectral Trend Observed by KMD Bubble Plots

**Figure 5:**
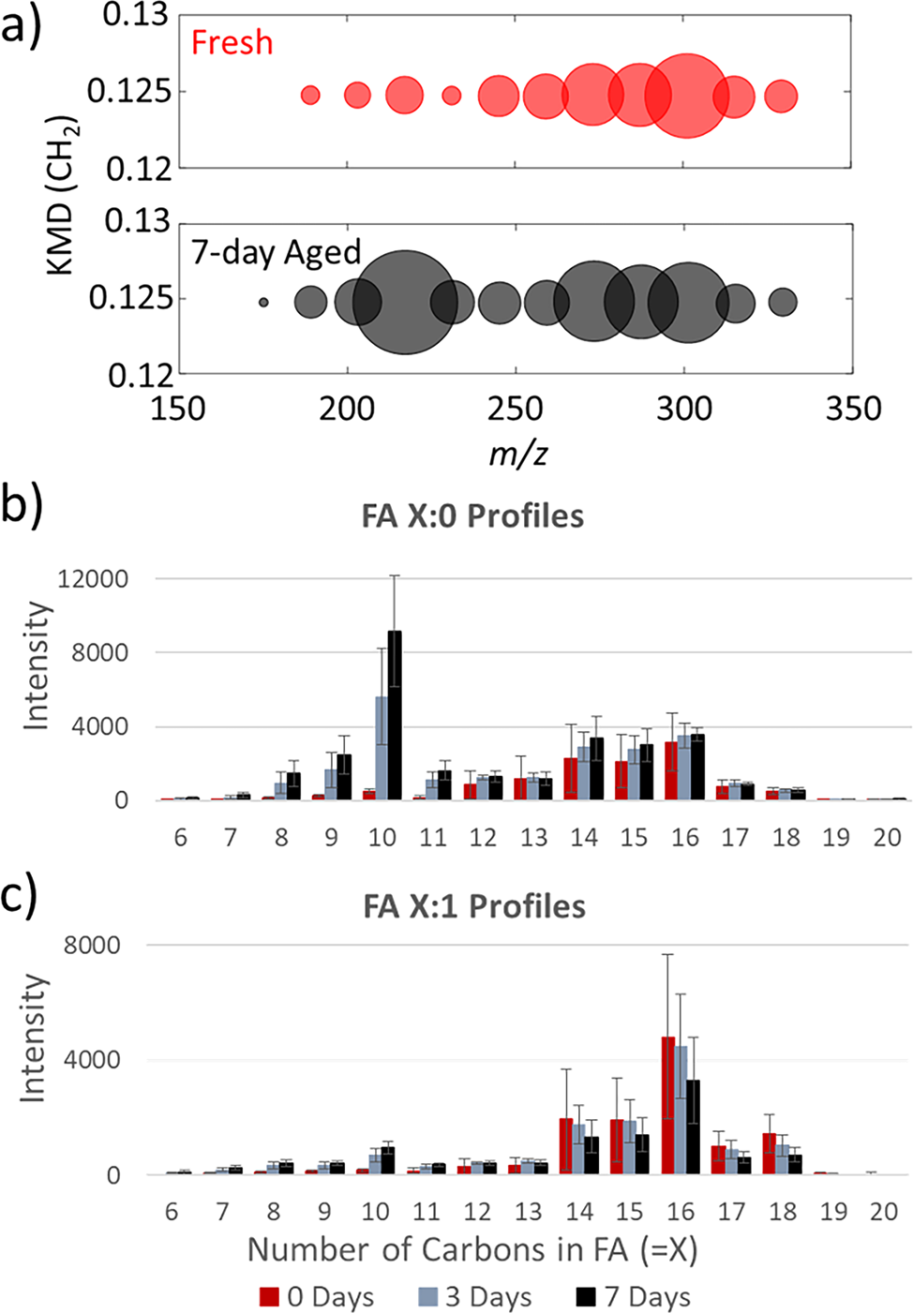
(a) KMD bubble plot for saturated fatty acids (C_c_H_2c-Z_O_2_Na_2_, DBE = 1) in fresh and 7-day aged fingerprints. Intensity profiles for (b) saturated and (c) monounsaturated FAs during fingerprint aging. Error bars represent one standard deviation from four replicates.

When assessing the intensity profile of the fatty acid heteroatom class (C_c_H_2c-Z_O_2_Na_2,_ red □ in Figure S5), one can see a stark increase in the intensity for *m*/*z* 217.117 in the KMD bubble plots after aging for 7 days (Figure 5a). This is a peak corresponding to FA10:0, which cannot be explained as a common contamination (e.g., vacuum pump oil), and saturated lipid species should not intuitively increase in signal due to aging. Additionally, the corresponding MS image demonstrates that the ion is localized to the ridge of the fingerprint (Figure S10a). We hypothesize that this is due to the ozonolysis of fatty acyl chains with a double bond at the ω-10 position (product **C** in Scheme 1). If an ω-10 fatty acyl chain is common in fingerprint lipids, ozonides at the ω-10 position will accumulate over time, resulting in an abundance of FA10:0 during MALDI analysis (product **C** in Scheme 1).

To demonstrate this possibility, an on-surface Paternò–Büchi (PB) reaction, using similar methodology from the literature,^37^ was performed to determine the double bond localization for a few sampled TGs in fresh fingerprints (see Figure S16 for the workflow). In the PB reaction, the lipid double bonds react with an aldehyde or a ketone reagent under UVC to form an oxetane ring. The derivatized lipid can be selected for MS/MS to produce diagnostic fragments for the double bond position. New peaks corresponding to the PB reaction of unsaturated TGs are observed in Figure S17b, compared to no reaction (Figure S17a), which are then subjected to MS/MS. As shown in Figure S17c for the MS/MS of derivatized TG 48:2 as an example, the most abundant double bond position is ω-10 on a 16:1 acyl chain. All MS/MS results are summarized in Table S3, and ω-10 is the most abundant in all TG species investigated (56–80%). The prevalence of ω-10 is also supported by the 10-carbon difference between the peak positions of TG(A) and TG along the *m/z* axis, TG 47:1 vs TG(A) 37:0 (Figure 3), as well as DG(A) and DG, DG 32:1 vs DG(A) 22:0 (Figure S15). Furthermore, sapienic acid (FA16:1, ω-10), though rare generally, is known to be a unique component of sebum.^38^ ESI-MS/MS of the FA(E)16:0 is consistent with the prevalence of sapienic acid in the fingerprints (Figure S11). Also, Pleik et al. reported decanal, originated from ω-10 FA, as the major fingerprint aging product in their GC-MS analysis^11^ and ω-10 as a common double bond position in fingerprint TGs in their LC-MS/MS of TG ozonides.^12^

Figure 5b,c shows the intensity profiles for saturated and monounsaturated FAs during aging, respectively. One can readily see the increase of FA 10:0 over time. It appears that ω-8 through ω-11 double bond positions also have some contribution, resulting in FA 8:0 through FA 11:0, which is consistent with the PB results (Table S3). These medium chain FAs (MCFAs), especially FA 10:0, may be good candidates to monitor for aging as they increase over time. In contrast, long chain FAs, FA 13:0 through FA18:0, are consistent regardless of aging because they are not oxidation fragments but mostly endogenous fatty acids. They might be useful for normalization when FA 10:0 is used for an aging marker, to account for differences in sebum loading during deposition. However, caution is required as these FAs might also come from contaminants. For example, 10% of FA 16:0 was from contamination in our experimental condition; hence, FA 15:0 or FA 17:0, with no or minimum contamination, might be better for normalization. It contrasts with MCFAs that are rare in nature, other than coconut, palm, and milk oils;^39^ thus, these features are very unlikely coming from contamination. A similar trend is found for monounsaturated FAs, especially FA 10:1 (Figure 5c), although ion intensity is much less. They are probably from polyunsaturated fingerprint lipids with an ω-10 as the second double bond position in a fatty acyl chain. The typical aging trend is found for FA 14:1 through FA 20:1, where intensity of the unsaturated signal decreases over time. It should be noted that FA 10:0 is mostly produced by in-source fragmentation of the secondary ozonide during MALDI (Scheme 1). Pleik et al. also observed FA 10:0 in their GC-MS analysis of aged fingerprints but in much lower abundance and attributed to the oxidation of decanal.^11^

A robust model for time since deposition should have multiple spectral features to describe different periods of the aging. In such a model, squalene oxidation may best describe early aging (<3 days), TG degradation captures midrange aging (2–7 days), and the accumulation of FA 10:0 has the potential to verify and extend the 7-day aging window (Figure S18). Future work will focus on kinetics-based and machine learning-based approaches to generate these models with larger data sets.

## Conclusions

Aged sebaceous fingerprints are composed of a complex lipid mixture and their oxidation products. In spite of many studies, there has been no comprehensive investigation of their degradation processes. By employing KMD plots for sebaceous fingerprint analysis, two novel discoveries were made. An oxygen atom addition to unsaturated lipids is found regardless of fingerprint lipid species. It is attributed to ambient epoxidation by singlet oxygen based on previous studies for ambient oxidation of standard lipids. It is further hypothesized that epoxidation occurs mostly on the top monolayer, which explains rapid epoxide formation but no further increase over time. Additionally, an unusual increase of FA 10:0 is found as fingerprints age, which is attributed to the Criegee ion resulting from the ozonolysis of an ω-10 fatty acyl chain. The double bond position is further verified with an on-surface Paternò–Büchi reaction, demonstrating that an ω-10 carbon–carbon double bond position is common in fingerprint TGs. The current study, using KMD plot analysis, leads to a better understanding of ambient oxidation processes in fingerprint aging and allows us to work toward developing a comprehensive model for time since deposition.

## Methods

### Fingerprint Collection and Ambient Aging

Fourteen groomed fingerprints were acquired from a single individual. The individual washed their hands thoroughly with water and soap followed by air drying. The thumb was then rubbed on the donor’s forehead and a fingerprint was placed on a precleaned glass slide. Fingerprints were aged in the ambient laboratory environment for 0, 1, 3, 5, and 7 days, where 0-, 3-, and 7-day samples were analyzed in quadruplicate, and only one sample was analyzed for the 1- and 5-day samples. Relative humidity and temperature were monitored using a hygrometer (Excursion-Trac; Fisher Scientific) at 30 min intervals, and ozone was monitored using a 106-L ozone monitor (Ozone Solutions; Hull, IA, USA) at 1 min intervals. Measured values over the 7-day period are reported in Figure S3 and Table S1.

### Fingerprint Sample Preparation

Fingerprints and standards were sprayed with 10 mM sodium acetate in methanol using a TM sprayer (HTX Technologies; Chapel Hill, NC, USA). A flow rate of 0.03 mL/min was used for a total of eight passes with 3 mm spacing in a crisscross pattern at a velocity of 1200 mm/min with a nitrogen gas pressure of 10 psi and nozzle temperature of 30 °C. Gold was then sputtered on top of the fingerprint for 20 s at 40 mA using a Cressington 108 auto Sputter Coater (Ted Pella; Redding, CA, USA).

### Mass Spectrometry Analysis and Data Processing

A QExactive HF (Thermo Finnigan; San Jose, CA, USA) with a MALDI/ESI injector (Spectroglyph; Kennewick, WA, USA)^40^ was used for positive mode MALDI-MS analysis with a mass resolution of 240,000 at *m*/*z* 200, maximum injection time of 492 ms, *m*/*z* range of 100–1200, and raster step of 50 μm. Xcalibur, an in-house Python script for the extraction of ions of interest, and MATLAB were used for data analysis.

### Supporting Experiments

Methods for the experiments to produce supporting figures can be found in the Supporting Information.

## Supporting Information Available

Additional experimental details and discussion, unsubtracted mass spectra, various KMD plots, summary of PB results, type-II isotopic overlap, MS images, MS/MS of epoxides and PB product, TG epoxide profiles in various conditions, separation of O_6_ homologous series, and promising aging features over time (PDF)
Data files used in the KMD plots and the PB table (ZIP)
Transparent Peer Review report available (PDF)
