# Supplementary material for: Novel Ambient Oxidation Trends in Fingerprint Aging Discovered by Kendrick Mass Defect Analysis
Source: ACS Cent Sci. 2022 Sep 21;8(9):1328–35. doi: 10.1021/acscentsci.2c00408 (PMC9523776; doi:10.1021/acscentsci.2c00408)
Supplement: Supplementary file 1 — oc2c00408_si_001.pdf [file oc2c00408_si_001.pdf]

Supporting information for:

Novel Ambient Oxidation Trends in Fingerprint Aging Discovered by Kendrick Mass Defect Analysis

Andrew E. Paulson and Young Jin Lee*

Department of Chemistry, Iowa State University, Ames, Iowa 50011

*Correspondence should be addressed to:

Email: yjlee@iastate.edu

Table of Contents

Supporting Methods (Pg 2-3)	
Standards aged in parallel to the fingerprints.	Pg 2
Epoxide ESI-MS/MS experiment.	Pg 2
Minimal ozone aging experiment.	Pg 2
Paternò–Büchi experiment and data analysis.	Pg 2-3
Supporting Discussion (Pg 3)	
KMD plot normalization techniques	Pg 3
Supporting Tables (Pg 4-6)	
Table S1. Summary of monitored ambient laboratory variables.	Pg 4
Table S2. Summary of heteroatom classes targeted in the study.	Pg 5
Table S3. Paternò–Büchi carbon-carbon double bond assignments of some TGs in fingerprints	Pg 6
Supporting Figures (Pg 7-21)	
Figure S1. Original mass spectrum for fresh and 7-day aged fingerprint	Pg 7
Figure S2. Overlain Kendrick plot for fresh and 7-day aged prints for the entire *m/z* and Kendrick mass defect range.	Pg 8
Figure S3. Monitoring the ambient laboratory variables over time.	Pg 8
Figure S4. Kendrick mass defect bubble plots.	Pg 9
Figure S5. Compound and heteroatom class annotated Kendrick plots.	Pg 10-11
Figure S6. Type-II spectral overlap example.	Pg 11
Figure S7. Dynamic normalization KMD plot example (x=9)	Pg12-13
Figure S8. KMD plots using a KMD base unit of $^{16}O_1$.	Pg 14
Figure S9. KMD plot of 7-day aged print using a KMD base unit of $^{12}C_1{}^{16}O_1$.	Pg 15
Figure S10. Mass spectrometry images of select ions related to fingerprint oxidation.	Pg 16
Figure S11. ESI-MS/MS spectrum of FA(E) 16:0 from fingerprint extract.	Pg 17
Figure S12. ESI-MS/MS spectrum of TG(E) 50:0 from aged standard extract.	Pg 17
Figure S13. Monitoring fingerprint lipid epoxide features during UVA aging experiment.	Pg 18
Figure S14. Monitoring fingerprint lipid epoxide features with different sample treatments.	Pg 18
Figure S15. Signal intensity profile of saturated DGs and the overlapped of TG, DG(A), and DG(E) series.	Pg 19
Figure S16. Paternò–Büchi experiment workflow.	Pg 20
Figure S17. Example MS and MS/MS spectra of derivatized fingerprint triacylglycerols	Pg 20
Figure S18. Compounds demonstrating the potential for time since deposition modeling.	Pg 21
Supporting References (Pg 21)	

SUPPORTING METHODS

Aging of Lipid Standards Preparation and Analysis. An equimolar mixture of standard TGs, 1.2 mM each of TG 54:3, TG 52:2, TG50:1, and TG48:0, where all carbon-carbon double bonds lie on 18:1 (ω-9) FA chain, was prepared in chloroform. The standard mixture was sprayed onto precleaned glass slides using a TM Sprayer (HTX Technologies; Chapel Hill, NC, USA). A flow rate of 0.03 mL/min was used for a total of eight passes with 3 mm spacing in a crisscross pattern at a velocity of 1200 mm/min with a nitrogen gas pressure of 10 psi and nozzle temperature of 30 °C. The resulting thin film of lipid standard was aged in the ambient lab conditions, in parallel to fingerprints, for 0, 1, 3, 5, or 7 days. Standards were prepared with sodium acetate and a gold sputter as described in the main text. Standards were analyzed by MALDI-MS with a QExactive HF (Thermo Finnigan; San Jose, CA, USA) equipped with a MALDI/ESI injector (Spectroglyph; Kennewick, WA, USA)[1] , using the same parameters described in the main text.

Epoxide ESI-MS/MS Experiments. Extracts of aged fingerprints and thin film TG standards were used in this experiment. A mixture of 0.12 mM TG 50:1 and TG 48:0 in chloroform was sprayed onto three glass slides (25x75x1mm) using the same TM Sprayer method described above except only four passes were applied. These standards were aged for 1 day in the ambient lab environment. The glass slides of the aged standard mixture or 3 fresh fingerprints deposited in 20 mL scintillation vials were extracted using 3 mL of the MeOH:$CHCl_3$ (1:1) doped with 5 mM of ammonium acetate to promote ionization in positive ion mode. Extracts were analyzed using the ESI source on the QExactive HF equipped with a MALDI/ESI injector. A flow rate of 5 µL/min and spray voltage of 3.5 kV was used for positive and negative mode. An isolation window of ±0.2 Da was used. A normalized collision energy (NCE) of 15 was used for FA analysis in negative mode and a NCE of 30 was used for TG analysis in positive mode. The results are summarized in **Figure S11** and **Figure S12**.

Controlled Fingerprint Aging under UVA. An ICH110L climate chamber (Memmert, Schwabach, Germany) was used to control relative humidity, temperature, and UVA light exposure (25.5%, 20.6°C, UVA light module on) during aging. Ozone concentration in the climate chamber was monitored using a 106-L ozone monitor (Ozone Solutions, Hull, Iowa, USA) at 1 min intervals. Samples were aged in the climate chamber for 3 days or placed in a desiccator in a closed container for 3 days (designated as fresh prints). The average ozone concentration over the 3 days was 2.7 ± 0.7 ppb, approximately a fifth of the ambient ozone concentration. The results are summarized in **Figure S13**. Fingerprint samples were prepared and analyzed by MALDI-MS using the same methods described in the main text.

On-surface Paternò–Büchi (PB) derivatization and sample preparation. The overall workflow is summarized in **Figure S16**. Benzophenone, 0.5 mL of 50 mM in chloroform, was sprayed onto the fingerprint using an oscillating spray device which was made by modifying a commercially available airbrush (Aztek A470; Testor, Rockford, IL, USA).[2] Briefly, the inner spraying tip was replaced with a fused silica capillary (150 µm i.d., 360 µm o.d.; Polymicro Technology, Phoenix, AZ, USA). Benzophenone solution was sprayed with a flow rate of 3.0 mL/hr using a syringe pump (11 Plus; Harvard Apparatus, Holliston, MA, USA). Nitrogen gas, the sheath gas, was fed through the airbrush at 38 psi. The sample was illuminated with 254 nm light using a UV lamp (3UV-38 3UV Lamp; UVP, Cambridge, UK) during benzophenone application. The UV lamp and the spray tip were located 12 cm above the sample surface. After derivatization, samples were prepared with the same matrix system described in the main text.

Paternò–Büchi Data Acquisition. MALDI-MS and MS/MS analysis were performed on a MALDI-LTQ-Orbitrap Discovery (Thermo Finnigan, San Jose, CA) with an external frequency tripled 355 nm Nd:YAG laser (UVFQ; Elforlight, Ltd., Daventry, UK). A 100 µm raster step, laser spot size of

approximately 5 μm, and ten laser shots in positive mode for the mass range of 100–1200 with a mass resolution setting of 30,000 at *m/z* 400 in the MS level. MS/MS was performed using the linear ion trap with a collision energy of 75 (arbitrary units) and an isolation width of 0.5–1.0 Da. The data was processed with Xcalibur.

Paternò–Büchi Data Analysis. Using benzophenone (BP) as a PB reagent for unsaturated lipids leads to a mass shift of 182.073 in the MS level (**Figure S17a** and **S17b**), indicating oxetane ring formation through [2+2] photocycloaddition. The mass shifted *m/z* ratios can then be selected for MS/MS experiments to determine the double bond site as summarized in **Table S3**. MS/MS of BP-derivatized TG 48:2 is shown in **Figure S17c** as an example. It should be noted that fingerprint TGs are composed of multiple combinations of fatty acyl chains and we cannot differentiate sn-1 vs sn-2 position. In addition to the neutral loss of FA 16:0 (*m/z* 751.6) and FA 16:1 (*m/z* 753.6), the neutral loss of a BP-derivatized FA 16:1 is found at *m/z* 571.5, indicating TG 48:2 is mostly composed of TG 16:1/16:1/16:0. When oxetane ring fragments via retro-cycloaddition during MS/MS, it results in the loss of benzophenone at m/z 825.7 and the two characteristic fragments with Δ*m/z* of 150.1, corresponding to the two orientations of benzophenone at the double bond site, "head-to-head" and "head-to-tail". The double bond site can be deduced from the neutral loss to the head-to-head peak being an aldehyde, $C_nH_{2n}O$. For example, the most abundant characteristic peaks for derivatized TG 48:2 are found at m/z 701.6 and 851.7, and the neutral losses correspond to n=10, thus ω-10. The other set of diagnostic fragments are marked as solid fill yellow and purple boxes in **Figure S17c** indicating the presence of ω-9 and ω-8, respectively.

SUPPORTING DISCUSSION

Describing undefined plot features. There are series in the KMD plots that were not annotated with the list described and not obviously explained by ozonolysis. Further finding unassigned trends can be aided by dynamic normalization, which is similar to fractional base units described by Fouquet *et al.*[3] **Figure S7** is an example of this normalization where KM is equivalent to the product of the *m/z* and x, user specified number, divided by the IUPAC exact mass of the Kendrick base unit. This method changes the vertical dispersion of different homologous series, while maintaining the horizontal alignment within a homologous series. This can allow for the interpretation of closely aligned series observed when using static normalization alone. Using the dynamic KMD plot with x = 9, ^{13}C isotope series could be successfully separated and annotated in **Figure S7.**

After accounting for ^{13}C isotopes, there are still some undefined series that need further investigation, some of which were investigated by the plots generated with different KMD base units. An example is using atomic oxygen, $^{16}O_1$ as a KMD base unit (**Figure S8**). Though the information is also in the CH_2 KMD plots, differences in oxygen content can easily be observed in this plot. We highlight two heteroatom classes that are more clearly observed in these plots. Including a series with high oxygen content in the fresh prints, which could be exogenous compounds (**Figure S8c**), and a heteroatom class of $C_cH_{2c-Z}ONa$ in the low mass range (*m/z* 250-300) which are tentatively assigned as unsaturated aldehydes (**Figure S8d**). Aldehyde species are expected to result from ozonolysis but the resulting peaks are not in the expected mass range. Instead, they are comprised of 16 to 18 carbons and are present in the both the aged and fresh fingerprints. A KMD plot using $^{12}C_1{}^{16}O_1$ as a base unit helps detect a cluster of features present primarily in the 7-day aged plot, but these features are not likely to be associated with aging as they are near the S/N>30 cutoff and are also present in the fresh spectrum at a similar S/N (**Figure S9**).

SUPPORTING TABLES

Table S1. Summary of the environmental conditions during the aging**.**

	1 day		3 day		5 day		7 day	
	Average	Std Dev	Average	Std Dev	Average	Std Dev	Average	Std Dev
**RH(%)**	30.3	1.7	30.3	10.1	26.7	9.1	25.3	8.6
**Temp (°C)**	20.6	0.2	20.7	0.2	20.6	0.2	20.6	0.2
**[Ozone] (ppb)**	6.7	2.5	12.7	5.1	13.8	4.8	13.3	5.1

Foot note: Values are calculated from **Figure S3**.

Table S2. Summary table of searched heteroatom classes expected from ozonolysis.

Heteroatom class	Substrate Species	Ozonolysis Substrate or Product	# of Acidic Hydrogens	# of H+ exchanged for Na+	Adduct	Z min	DBE min	Searched DBE values	Symbol in Figure 2 & S3
$C_cH_{2c-z}O_2Na$	WE	Substrate	0	0	Na	0	1	1, 2, 3, 4, 5, 6	□
	FA	Substrate	1	0	Na	0	1	1, 2, 3, 4, 5, 6	
$C_cH_{2c-z}O_2Na_2$	FA	Substrate	1	1	Na	1	1.5	1.5, 2.5, 3.5, 4.5	□
$C_cH_{2c-z}O_3Na$	WE	A	0	0	Na	2	2	2, 3, 4, 5	◊
	FA	A	1	0	Na	2	2	2, 3, 4, 5	
$C_cH_{2c-z}O_3Na_2$	FA	A	1	1	Na	3	2.5	2.5, 3.5, 4.5	◊
$C_cH_{2c-z}O_4Na$	WE	B/C	0/1	0	Na	2	2	2, 3, 4, 5	◊
	FA	B/C	1/2	0	Na	2	2	2, 3, 4, 5	
	WE	AA	0	0	Na	4	3	3, 4, 5	
$C_cH_{2c-z}O_4Na_2$	WE	C	1	1	Na	3	2.5	2.5, 3.5, 4.5, 5.5	◊
	FA	B/C	1/2	1	Na	3	2.5	2.5, 3.5, 4.5, 5.5	
$C_cH_{2c-z}O_4Na_3$	FA	C	2	2	Na	4	3	3, 4, 5	◊
$C_cH_{2c-z}O_5Na$	DG	Substrate	0	0	Na	2	2	2, 3, 4, 5	□
	WE	AB/AC	0/1	0	Na	4	3	3, 4, 5	
$C_cH_{2c-z}O_5Na_2$	WE	AC	1	1	Na	5	3.5	3.5, 4.5, 5.5	Δ
$C_cH_{2c-z}O_6Na$	TG	Substrate	0	0	Na	4	3	3, 4, 5, 6, 7	□
	DG	A	0	0	Na	4	3	3, 4, 5, 6, 7	
	WE	BB/BC/CC	0/1/2	0	Na	4	3	3, 4, 5, 6, 7	
$C_cH_{2c-z}O_6Na_2$	WE	BC/CC	1/2	1	Na	5	3.5	3.5, 4.5, 5.5	Δ
$C_cH_{2c-z}O_6Na_3$	WE	CC	2	2	Na	6	4	4, 5, 6	x
$C_cH_{2c-z}O_7Na$	TG	A	0	0	Na	6	4	4, 5, 6, 7	◊
	DG	B/C	0/1	0	Na	4	3	3, 4, 5, 6, 7	
	DG	AA	0	0	Na	6	4	4, 5, 6, 7	
$C_cH_{2c-z}O_7Na_2$	DG	C	1	1	Na	5	3.5	3.5, 4.5, 5.5	x
$C_cH_{2c-z}O_8Na$	TG	B/C	0/1	0	Na	6	4	4, 5, 6, 7	◊
	TG	AA	0	0	Na	8	5	5, 6, 7	
	DG	AB/AC	0/1	0	Na	6	4	4, 5, 6, 7	
$C_cH_{2c-z}O_8Na_2$	TG	C	1	1	Na	7	4.5	4.5, 5.5, 6.5, 7.5	◊
	DG	AC	1	1	Na	7	4.5	4.5, 5.5, 6.5, 7.5	
$C_cH_{2c-z}O_9Na$	TG	AB/AC	0/1	0	Na	8	5	5, 6, 7	x
	DG	BB/BC/CC	0/1/2	0	Na	6	4	4, 5, 6, 7	
$C_cH_{2c-z}O_9Na_2$	TG	AC	1	1	Na	9	5.5	5.5, 6.5, 7.5	Δ
	DG	BC/CC	1/2	1	Na	7	4.5	4.5, 5.5, 6.5, 7.5	
$C_cH_{2c-z}O_9Na_3$	DG	CC	2	2	Na	8	5	5, 6	x
$C_cH_{2c-z}O_{10}Na$	TG	BB/BC/CC	0/1/2	0	Na	8	5	5, 6, 7	x
$C_cH_{2c-z}O_{10}Na_2$	TG	BC/CC	1/2	1	Na	9	5.5	5.5, 6.5, 7.5	x
$C_cH_{2c-z}O_{10}Na_3$	TG	CC	2	2	Na	10	6	6, 7, 8	x

Foot notes for Table S2:

1. Not all searched heteroatom classes are found in KMD plot. "x" indicates searched theoretical heteroatom classes that were not found in the 7-day aged fingerprint in the lipid range for the KMD.
2. Several lipid substrates or their oxidation products belong to the same heteroatom class. The compounds within a heteroatom class are listed in order of expected relative signal contribution.
3. Z is an odd value for disodiated heteroatom classes and even otherwise. It increases by 2 for each DBE increase.

Table S3. Double bond assignments of selected fingerprint TGs by on-surface Paternò-Büchi reaction and MS/MS.

TG	Derivatized *m/z*	Characteristic fragments[a]	DB position[b]
45:1	967.732	811.7/661.5	**ω - 10 (56.5%)**
		839.7/689.6	ω - 8 (26.0%)
		825.7/675.6	ω -9 (17.5%)
46:1	981.747	825.6/675.5	**ω - 10 (69.9%)**
		853.7/703.6	ω - 8 (16.5%)
		839.7/689.6	ω -9 (13.6%)
47:1	995.761	839.6/689.6	**ω - 10 (59.3%)**
		853.7/703.6	ω - 9 (13.1%)
		867.8/717.6	ω -8 (10.9%)
		825.6/675.7	ω - 11 (8.8%)
		937.8/787.6	ω - 3 (7.9%)
48:2	1007.762	851.7/701.6	**ω - 10 (77.8%)**
		865.7/715.6	ω - 9 (11.1%)
		879.7/729.6	ω -8 (11.1%)
48:1	1009.778	853.7/703.6	**ω - 10 (80.8%)**
		867.6/717.6	ω - 9 (10.7%)
		881.8/731.6	ω - 8 (8.5%)
49:1	1023.782	867.7/717.6	**ω - 10 (67.9%)**
		853.7/703.6	ω - 11 (12.3%)
		881.7/731.6	ω - 9 (11.5%)
		895.7/745.6	ω - 8 (8.3%)
50:2	1035.788	879.7/729.6	**ω - 10 (79.7%)**
		865.7/715.6	ω - 11 (10.6%)
		893.7/743.7	ω - 9 (9.7%)

Foot notes for Table S3:

a. Fragment ion pairs with an aldehyde fragment signal intensity greater than 10% of the most abundant aldehyde fragment (often the base peak) are considered in order to avoid over interpretation. The colors in TG 48:2 correlate with the fragments in the example MS/MS spectrum shown in **Figure S17c**.

b. The most common double bond position is highlighted in green for each TG. The percentage in parenthesis is calculated from the summed intensity of each characteristic fragment pair.

SUPPORTING FIGURES

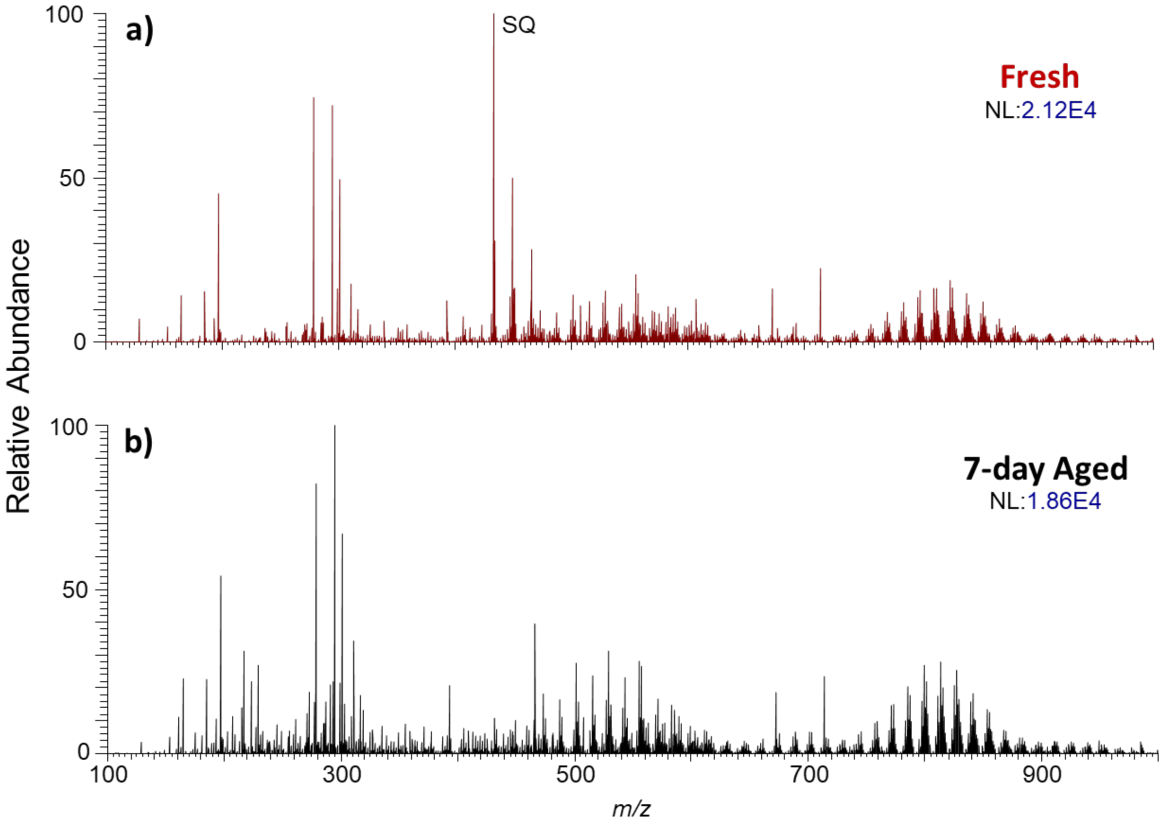

Figure S1. Mass spectrum of the (a) fresh and (b) 7-day aged fingerprints used for subtracted spectrum in **Figure 1**.

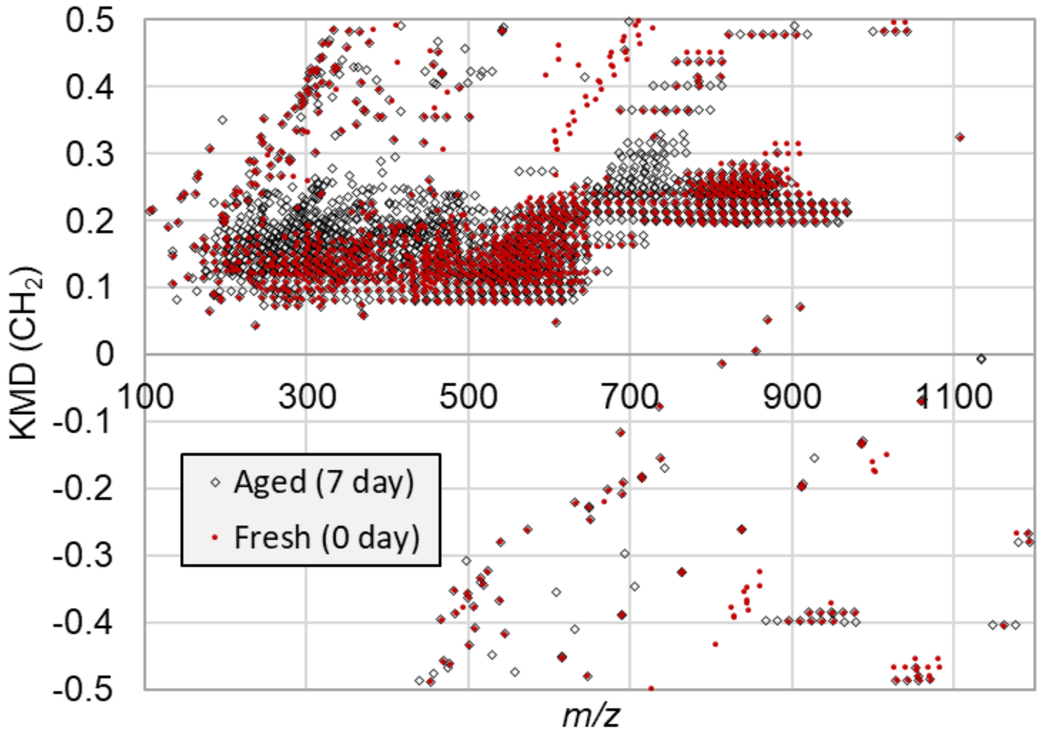

Figure S2. Overlain KMD plot of the fresh (red circle) and 7-day old (black diamond) fingerprints for the entire *m/z* and KMD range with S/N>30. Most peaks outside of the lipid KMD range are contamination.

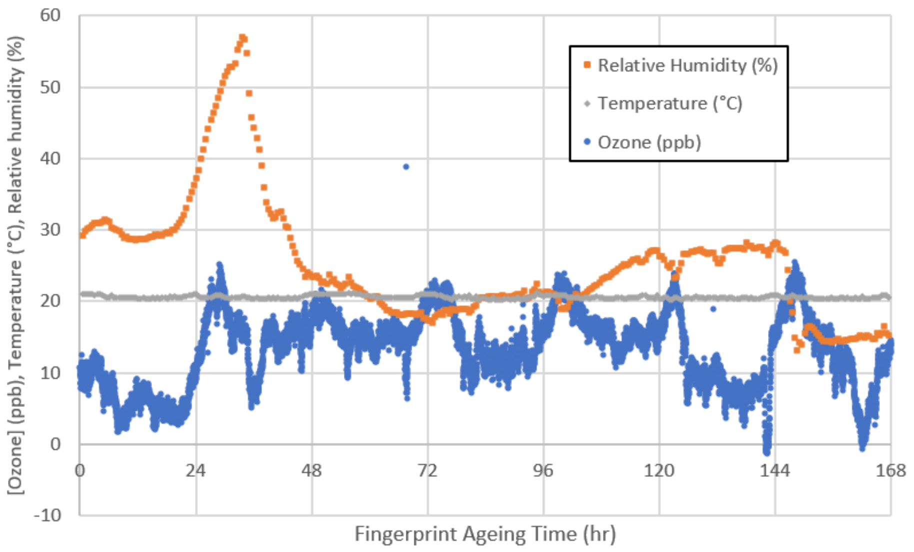

Figure S3. Monitoring the ambient laboratory variables over time. Relative humidity, temperature and ozone concentrations were monitored during the seven days of fingerprint aging.

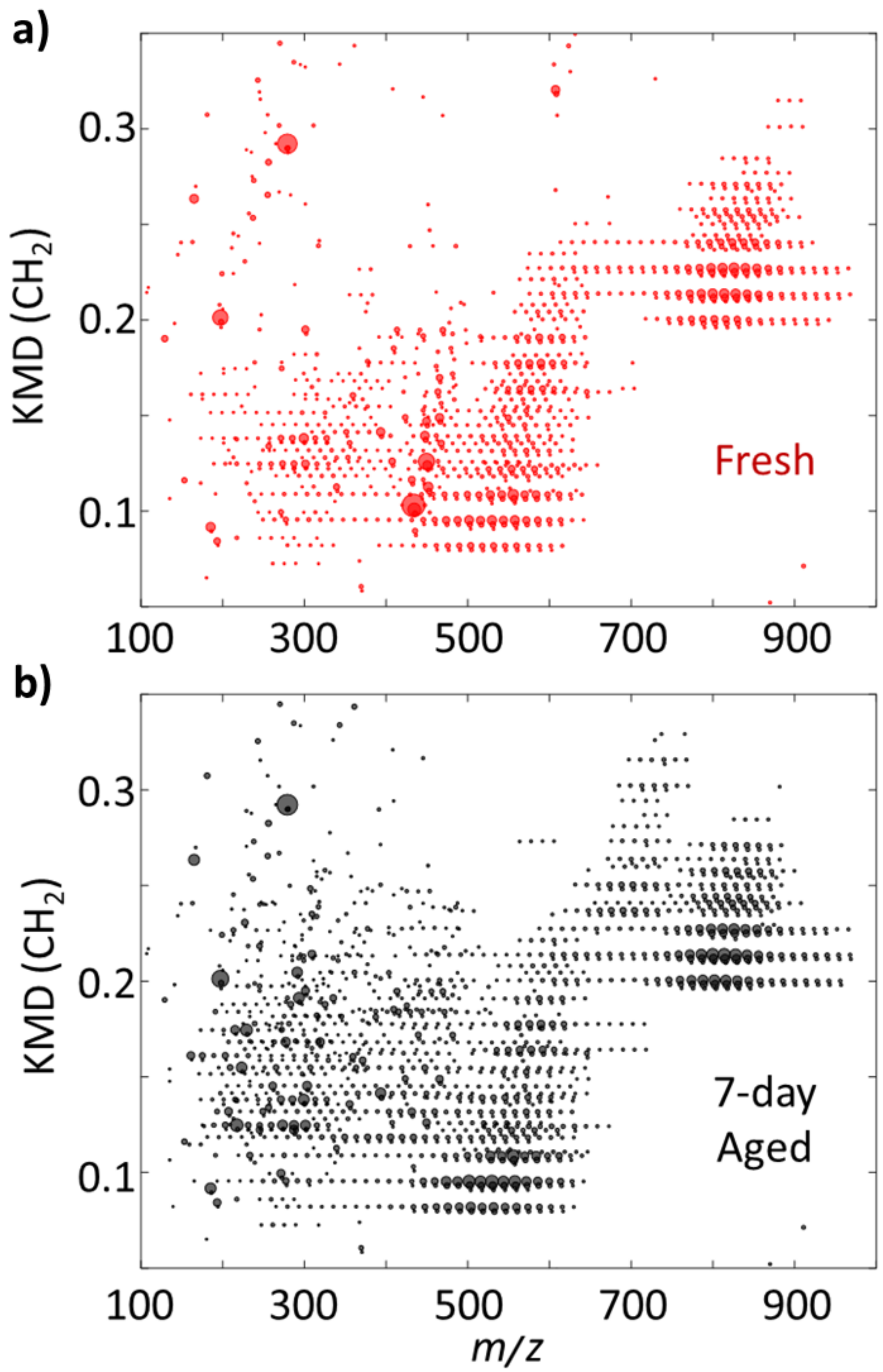

Figure S4. Kendrick mass defect bubble plots of the fresh (red) and 7-day old (black) fingerprints where bubble size is proportional to the relative abundance as seen in **Figure S1**.

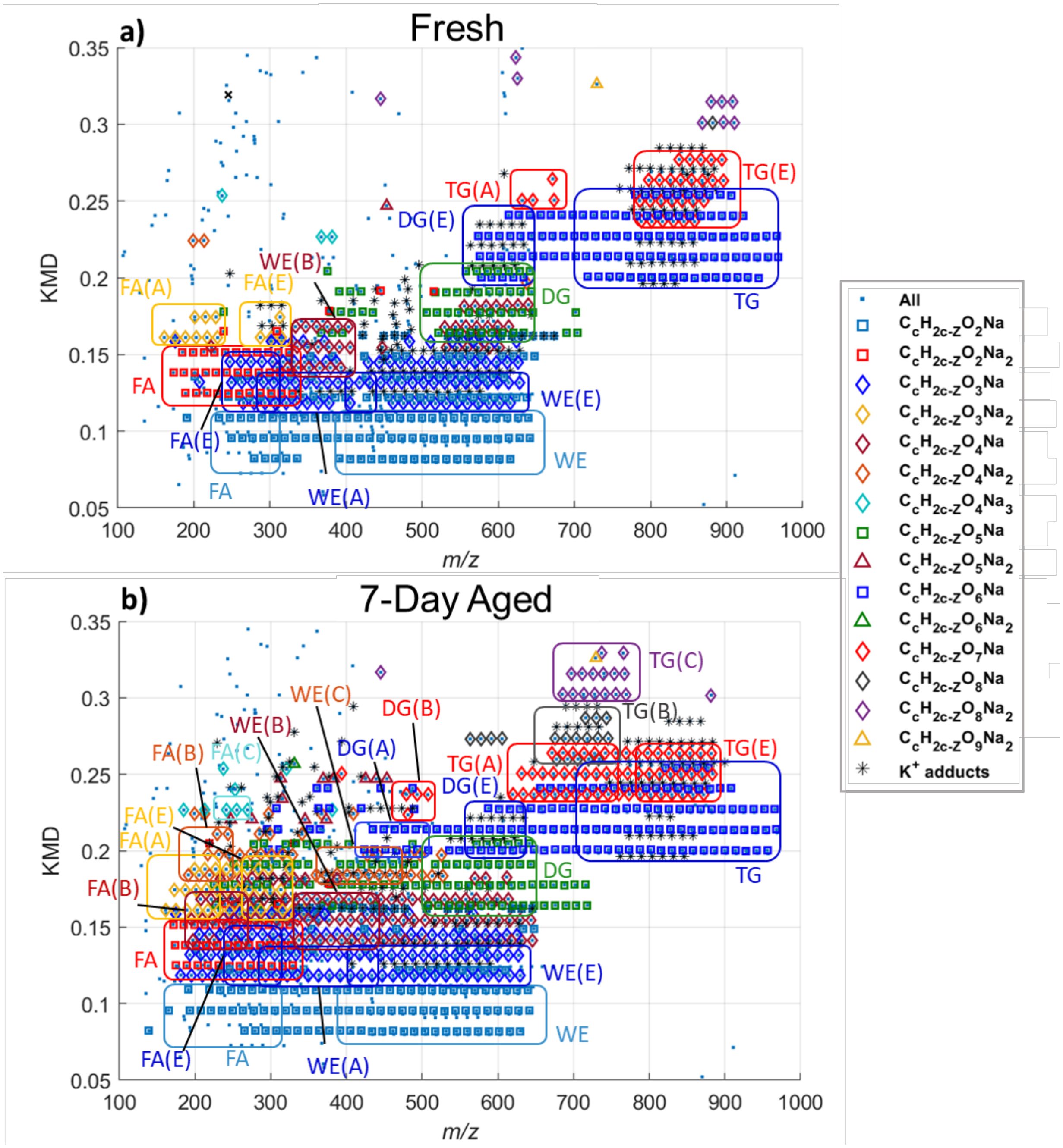

Figure S5. KMD plots for the **(a)** fresh and **(b)** 7-days aged fingerprint of the lipid region with S/N>30. Heteroatom class annotations are based solely on theoretical values in ambient ozonolysis (**Scheme 1**). The legend is based on searched heteroatom class summarized in **Table S2**. Tentative compound assignments are provided for undegraded precursors, a single ozonolysis process, and a proposed epoxidation process.

Foot notes for Figure S5:

1. To minimize the clutter, only the first ozonolysis or epoxidation product is annotated; e.g., TG(AA) is not shown. The two Criegee ions in **Scheme 1** (**B** and **C** product) cannot be distinguished when mono-sodiated and are labeled as '(B)', to simplify, instead of '(B/C)' as in **Figure 2**. Di-sodiated Criegee ion is only possible for carboxylic acid and labeled as '(C)'.
2. It is challenging to distinguish compounds within the same heteroatom class or those with very close KMDs. As long as they do not entirely overlap in the *m/z* dimension (e.g., **Figure 3**), such conflicts can be distinguished in a KMD plot.
3. Tentative compound assignments are made conservatively to minimize misinterpretation and a signal cutoff of S/N > 30 is used to avoid the overinterpretation of low intensity peaks.
4. Di-sodiated adduct of TG(C) (purple ◊) has very close KMD with the mono-sodiated TG(B) (black ◊). However, these series only overlap when interpreting three or more unsaturation in TG(B). Given that one of the double bonds in the original lipid must be consumed in the ozonolysis reaction to produce TG(B), the interference to the di-sodiated adduct profile is negligible. The same arguments can be made for the same ozonolysis products of the other lipid precursors.
5. Epoxides and aldehyde ozonolysis products have the same heteroatom class and cannot be distinguished.
6. We include $^{13}C_1$ and $^{13}C_2$ peaks in the KMD plots and include potassium adduct annotations to ensure that heteroatom class assignment is as accurate and transparent as possible. Some overlap is observed due to very close KMD with other compounds, but they are mostly distinguishable.

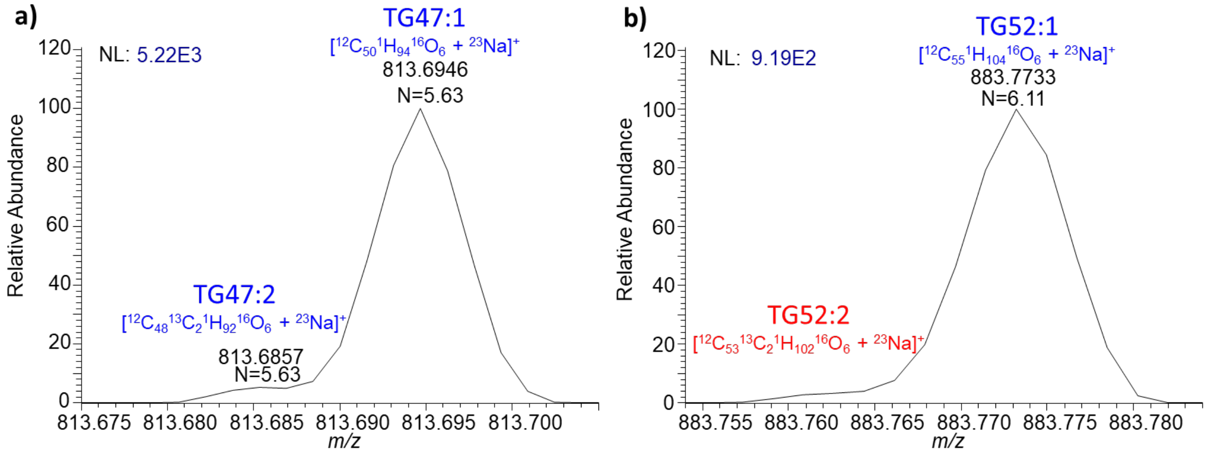

Figure S6. Type-II isotopic overlap within the 7-day aged fingerprint spectrum demonstrating some limitations of the instrumentation at a higher *m/z* range, especially with drastic signal differences.

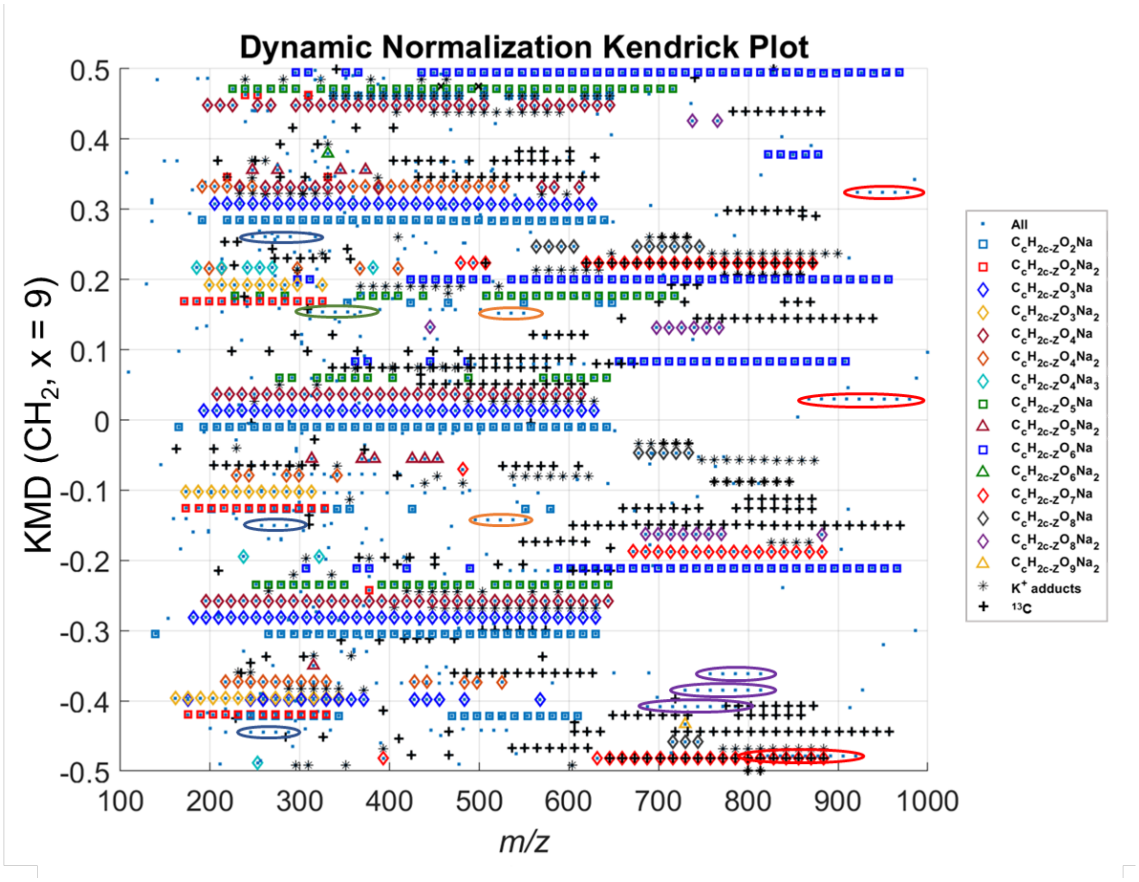

Figure S7. Dynamic normalization KMD plot of the 7-day aged fingerprint with x=9.

Foot notes for Figure S7:

1. ^{13}C isotope contributions, up to three ^{13}C atoms for all searched *m/z* values, were included and annotated in this plot.

2. The dynamic normalization uses a different calculation for the KMD:

   KMD = *m/z* * x/(14.01565)

   Instead of x=14, the user inputs other values in order to manipulate the vertical dispersion of different homologous series while retaining the horizontal alignment within a homologous series. This allows visualizing homologous series that are nearly overlapped with static, x=14, normalization.

3. Note that a majority of the plot features are defined. Most of the unassigned features are well separated in the x=14 plot, and are aliased into the KMD range of the defined lipids when dynamic normalization is used (features circled red). However, this plot serves to check to ensure homologous series are not overlooked. The relationships of heteroatom compositions of unassigned features can be aided with using a different KMD base, as described in Figure S11 and S12.

4. The features circled in blue are tentatively assigned as sodiated adducts of unsaturated aldehydes, $C_cH_{2c-Z}ONa$ (Z_{min} = 2), and those circled in green as sodiated adducts of monoacylglycerols, $C_cH_{2c-Z}O_4Na$ (Z_{min} = 0), where assignments were assisted by using a KMD base of O in Figure S11.

5. The features circles in purple are an unassigned heteroatom classes that differ by CO, as seen in Figure S12.

6. The features circles in orange are an unassigned heteroatom class with exact mass consistent with $C_cH_{2c-Z}O_4$ (Z_{min} = 2), as protonated adducts.

7. Note the misassignment of the $C_cH_{2c-Z}O_7Na$ features as the ^{13}C isotope peaks. This is an obvious misassignment considering the lack of monoisotopic peaks.

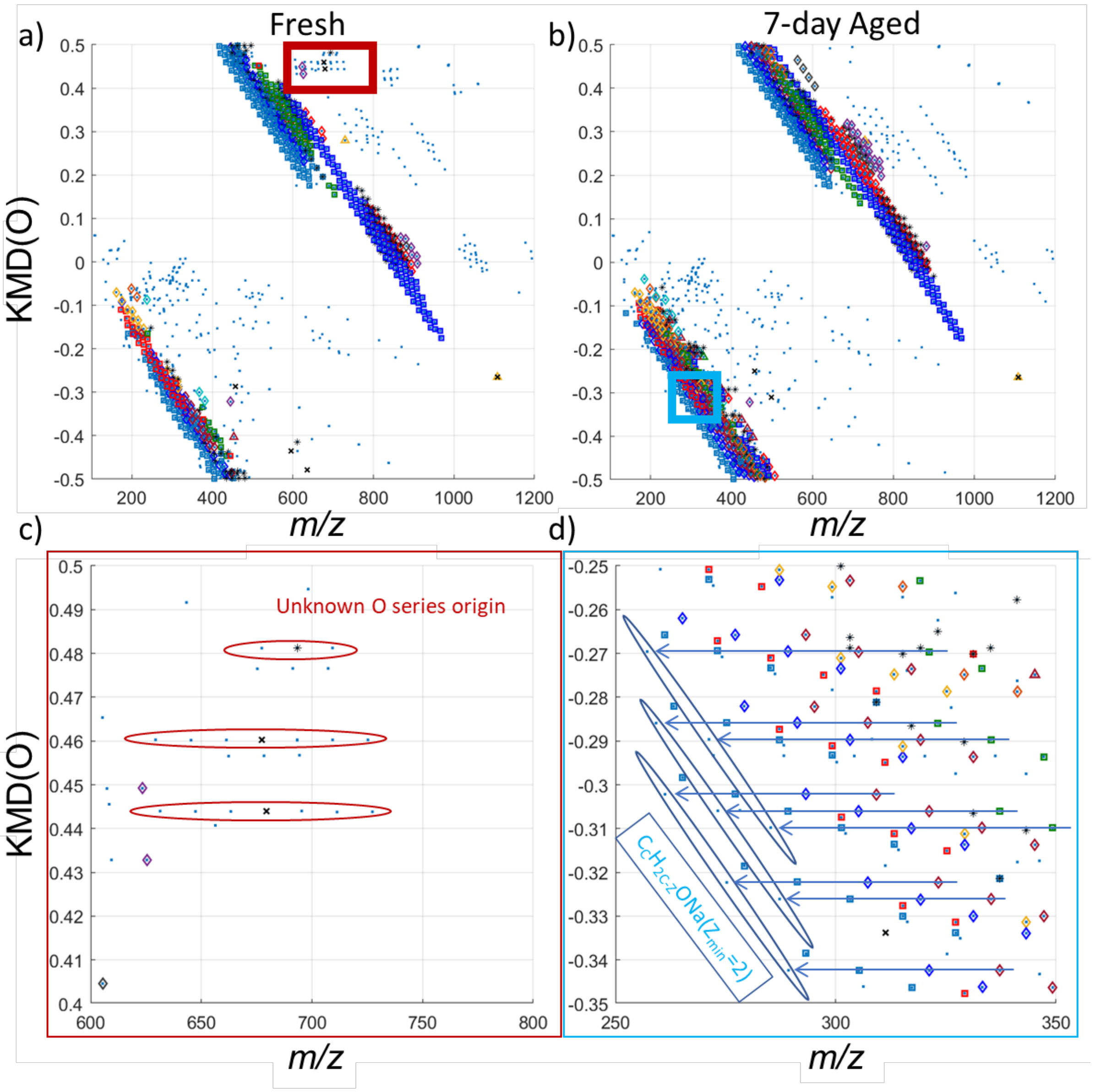

Figure S8. KMD plots using ^{16}O as a Kendrick mass base unit for the **(a)** fresh and **(b)** 7-day aged fingerprints. Horizontal alignments helped assign the elemental compositions of **(c)** a group of plot features differing by oxygen content present in the fresh print that have an unknown origin, and **(d)** a heteroatom class tentatively assigned as unsaturated aldehydes that was previously unassigned.

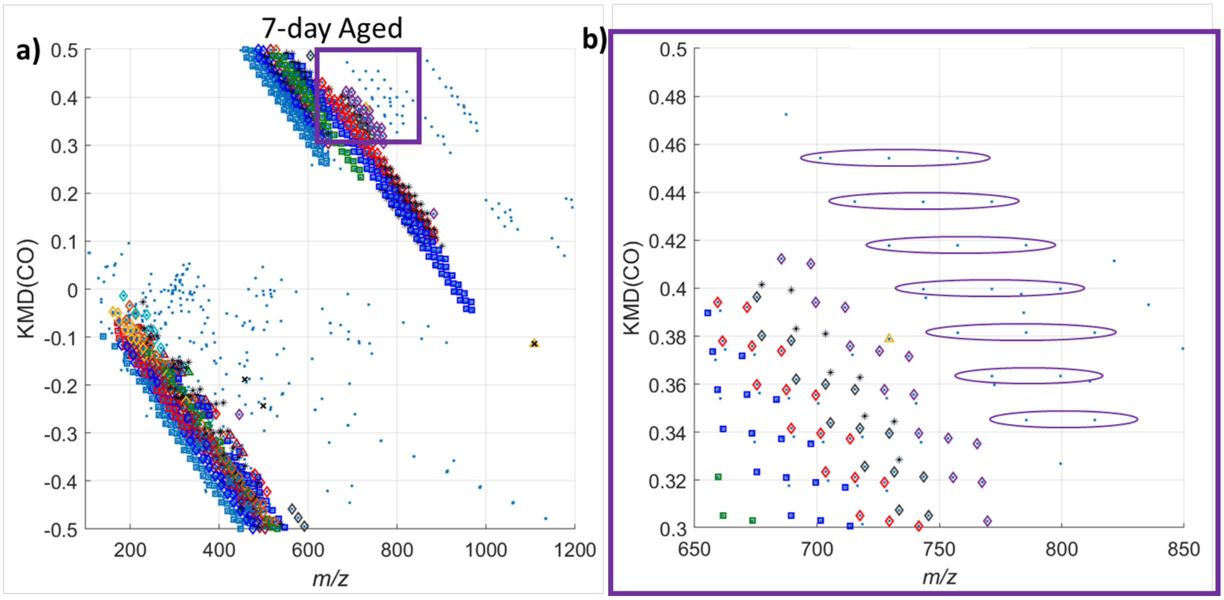

Figure S9. KMD plots using $^{12}C_1{}^{16}O_1$ as a Kendrick mass base unit for the **(a)** 7-day aged fingerprints. Horizontal alignments helped visualize **(b)** a group of unassigned plot features differing by CO oxygen content present in the 7-day aged fingerprint, though the origin is unknown.

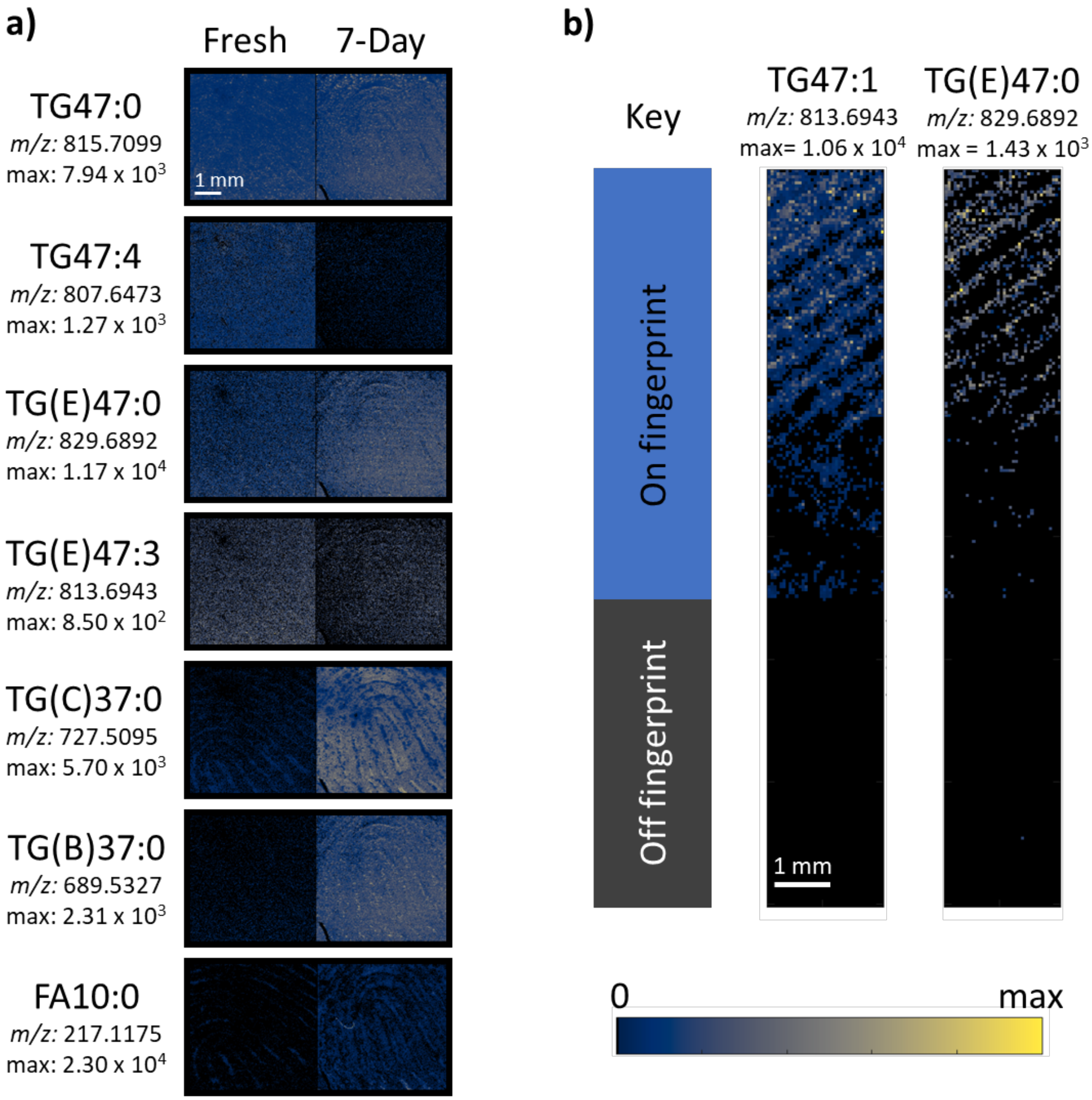

Figure S10. (a) Mass spectrometry images of a selection of *m/z* values (± 2 ppm) of interest for a section in the middle of a fresh and 7-day aged fingerprint (20 µm raster step). (b) Mass spectrometry images of TG47:1 and TG(E)47:0 at the edge of a fresh fingerprint (50 µm raster step). All adducts are $[M+Na]^+$ except TG(C)37:0 and FA10:0, which are $[M-H+2Na]^+$.

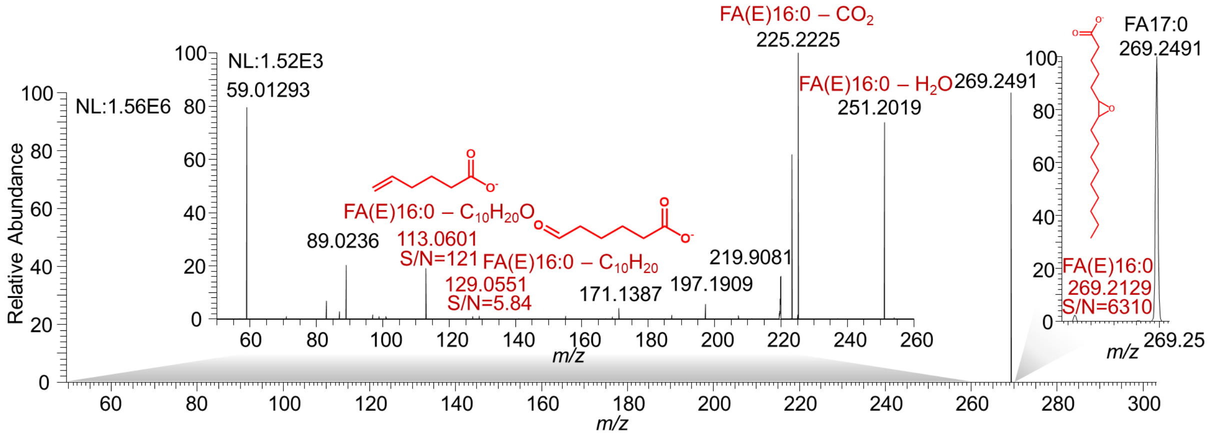

Figure S11. Direct infusion ESI-MS/MS of FA(E)16:0, $[M-H]^-$, from extracted fresh fingerprints. FA17:0, *m/z* 269.2491, is a co-isolated ion that is not expected to undergo significant fragmentation. A NCE of 30 is used. The mass error is less than ± 7 ppm for all assignments. The larger mass error tolerance is due to the poor calibration of *m/z* values below the *m/z* of dodecyl sulfate (*m/z* 265.1479), the lowest value used for negative mode calibration.

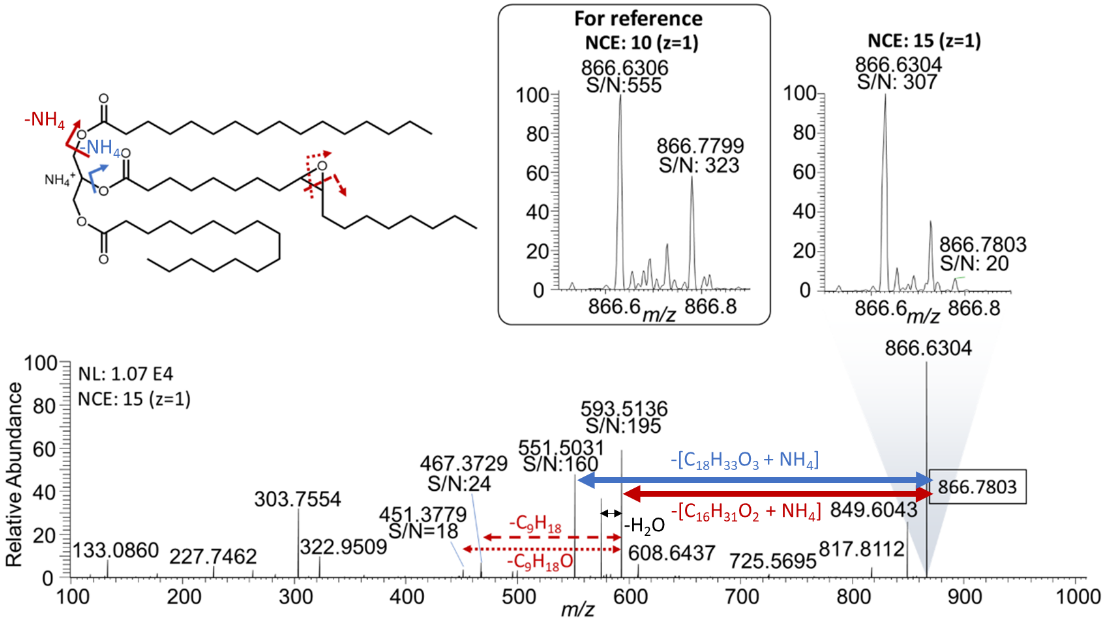

Figure S12. Direct infusion ESI-MS/MS of TG(E)50:0, $[M+NH_4]^+$, from an extracted thin film (~ 8 nm) of TG standard mixture (1:1, TG 50:1: TG48:0) aged in ambient laboratory conditions for one day. The thin film was extracted with MeOH:$CHCl_3$ (1:1) doped with 5 mM of ammonium acetate to promote ionization. Arrows in the structure indicate fragmentation channels corresponding to the major fragments in the MS/MS spectrum.[4] Dotted and dashed arrows are characteristic of epoxide fragmentation occurring in parallel to FA16:0 acyl chain fragmentation. A NCE of 15 is used for the theoretical *m/z* 866.7807 with the isolation window of ± 0.2 Da. There is a co-isolated interference ion at *m/z* 866.6304 but it does not appear to undergo significant fragmentation compared to *m/z* 866.7807 as shown in the inset zoomed-in spectra for the precursor at NCE of 10 and 15.

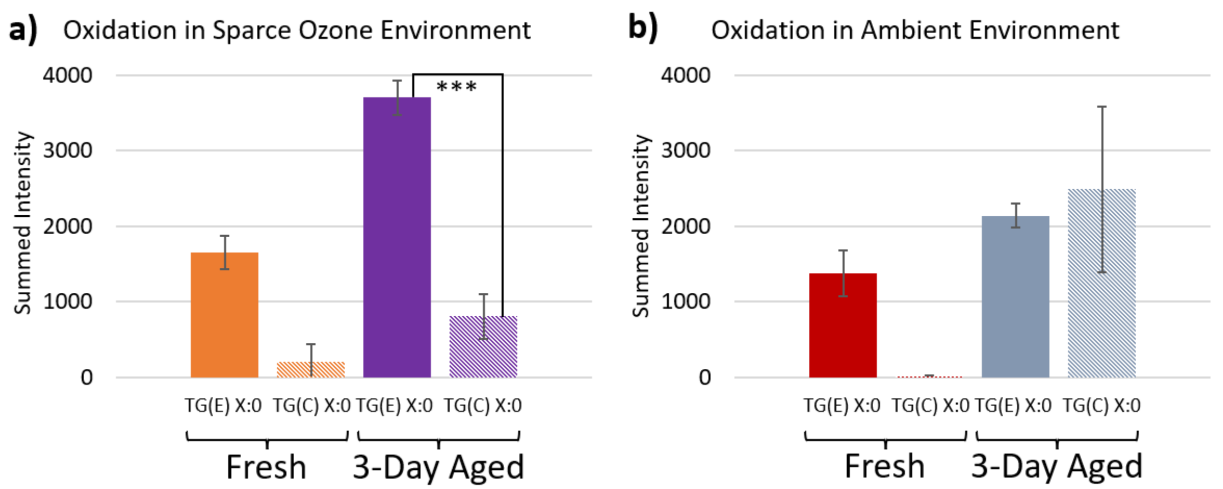

Figure S13. Comparing the summed signal from the saturated TG epoxide, TG(E) X:0, vs saturated TG carboxylic acid ozonolysis product, TG(C) X:0, during fingerprint aging **(a)** in a climate control chamber for 3 days with a UVA light on and **(b)** in the ambient lab condition. TG(C) is used instead of TG(A) as a representation of ozonolysis product because some TG(A) might have come from singlet oxygen according to Zhou et al.; however, the overall trend is the same. Error bars represent one standard deviation of four replicates. ***: $p < 0.001$

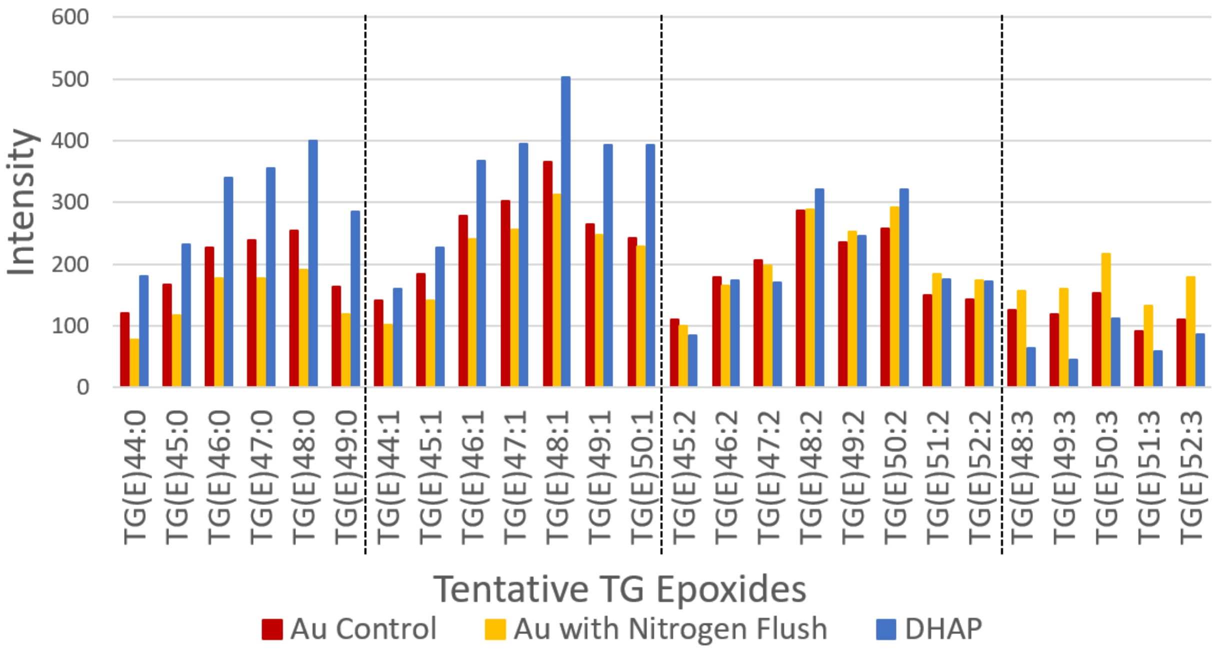

Figure S14. Test potential experimental artifacts in epoxide formation. Signal intensity of TG epoxides for fresh fingerprints in normal condition (Au Control) compared with pure nitrogen flush and the use of 2,5-dihydroxyacetophenone (DHAP) as a MALDI matrix.

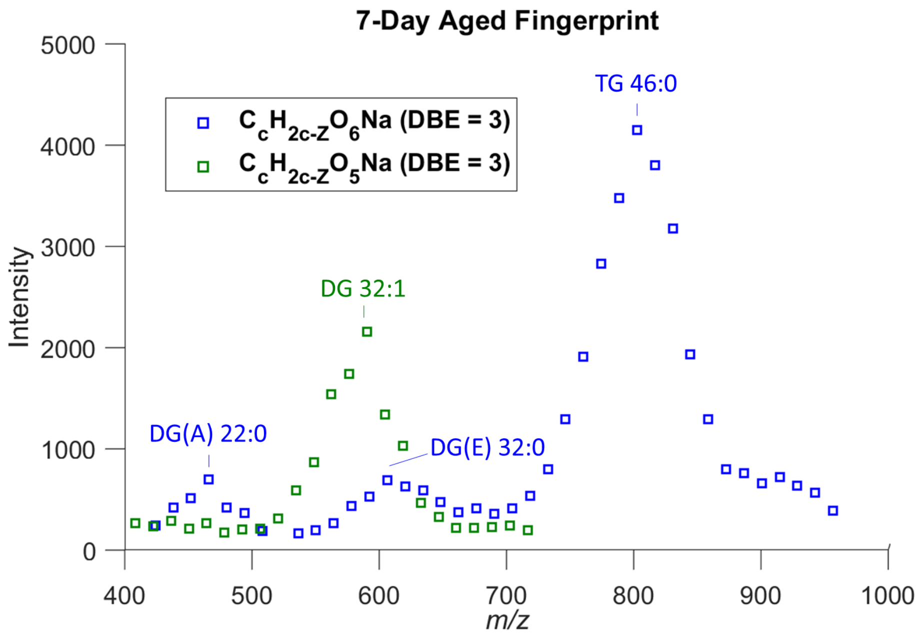

Figure S15. Intensity profile for $C_cH_{2c-Z}O_6Na$ (DBE=3) and $C_cH_{2c-Z}O_5Na$ (DBE=3) homologous series in the 7-day aged fingerprint.

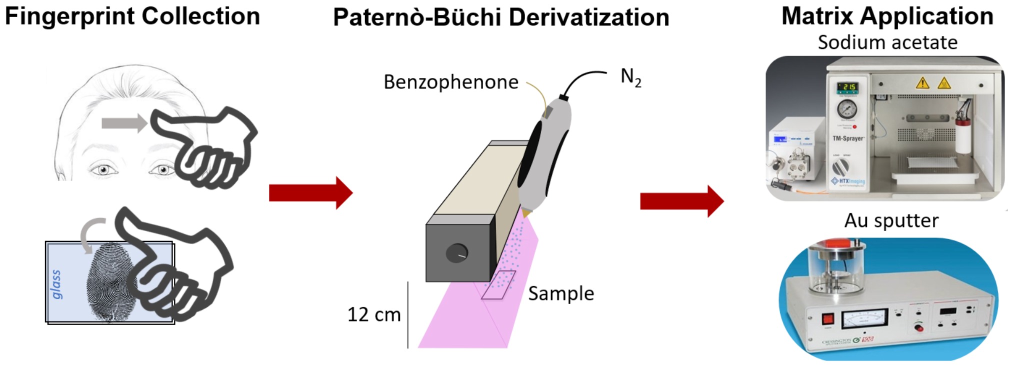

Figure S16. Workflow for Paternò–Büchi derivatization of fingerprints

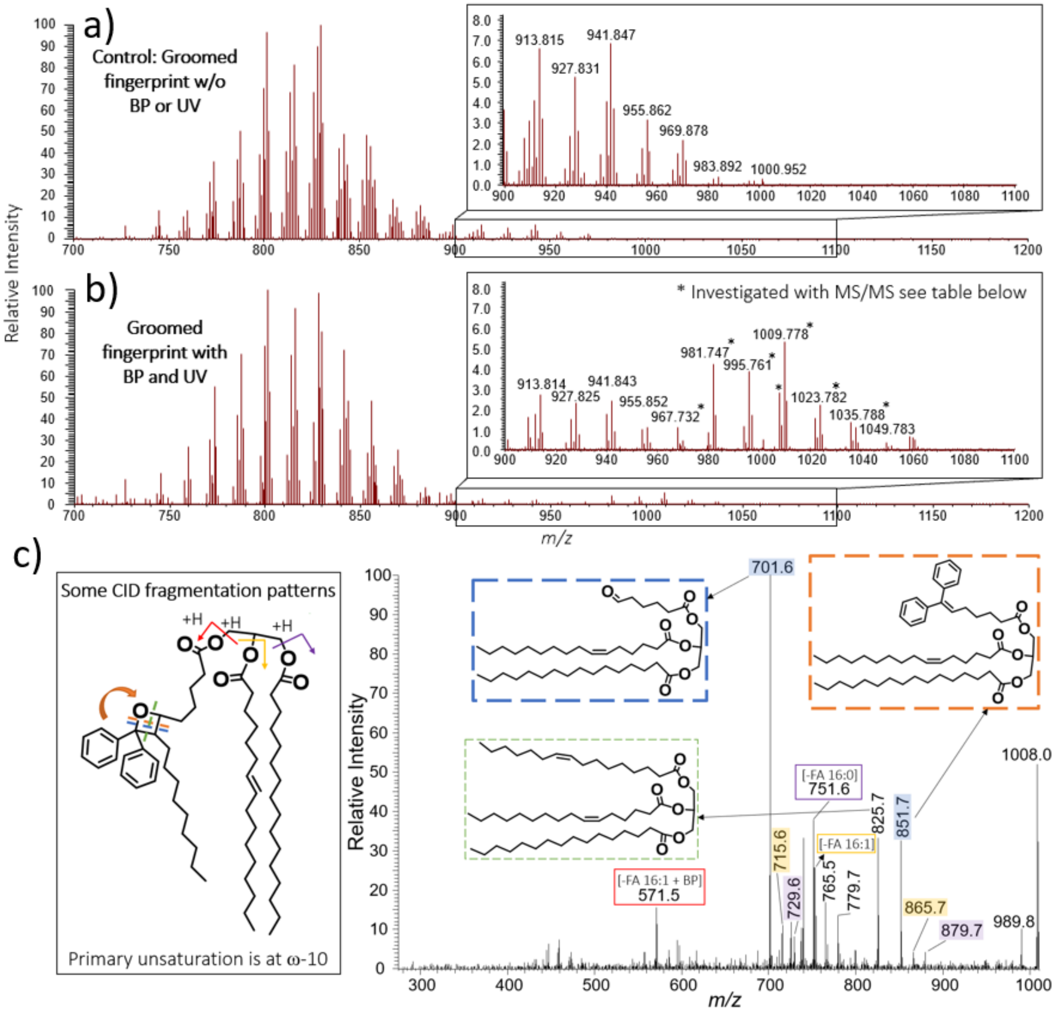

Figure S17. MALDI mass spectrum of TGs in a fresh fingerprint (a) without and (b) with the Paternò-Büchi reaction. Some of the new peaks corresponding to PB reaction products, asterisked in the inset spectrum of **Figure S17b**, were selected for MS/MS. (c) MS/MS spectrum of derivatized TG 48:2 as an example. *m/z* 701.6 and 851.7 are key fragments to indicate the double bond at ω-10 position.

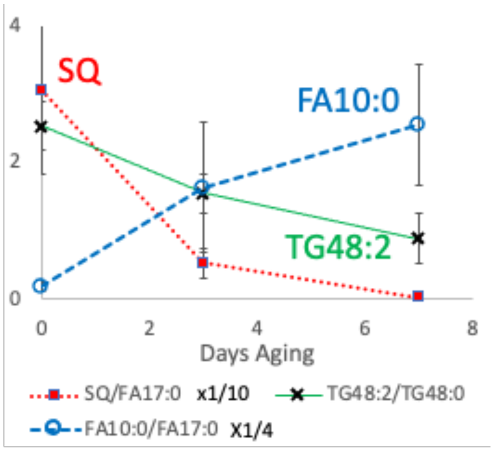

Figure S18. Temporal trends of promising targets for fingerprint time-since-deposition models.

References

(1) Belov, M. E.; Ellis, S. R.; Dilillo, M.; Paine, M. R. L.; Danielson, W. F.; Anderson, G. A.; de Graaf, E. L.; Eijkel, G. B.; Heeren, R. M. A.; McDonnell, L. A. Design and Performance of a Novel Interface for Combined Matrix-Assisted Laser Desorption Ionization at Elevated Pressure and Electrospray Ionization with Orbitrap Mass Spectrometry. *Anal. Chem.* **2017**, *89* (14), 7493–7501, DOI: 10.1021/acs.analchem.7b01168.

(2) Korte, A. R.; Yagnik, G. B.; Feenstra, A. D.; Lee, Y. J. Multiplex MALDI-MS Imaging of Plant Metabolites Using a Hybrid MS System. In *Mass Spectrometry Imaging of Small Molecules*; He, L., Ed.; Methods in Molecular Biology; Springer New York: New York, NY, 2015; Vol. 1203, pp 49–62, DOI: 10.1007/978-1-4939-1357-2_6.

(3) Fouquet, T.; Sato, H. Extension of the Kendrick Mass Defect Analysis of Homopolymers to Low Resolution and High Mass Range Mass Spectra Using Fractional Base Units. *Anal. Chem.* **2017**, *89* (5), 2682–2686, DOI: 10.1021/acs.analchem.6b05136.

(4) McAnoy, A. M.; Wu, C. C.; Murphy, R. C. Direct Qualitative Analysis of Triacylglycerols by Electrospray Mass Spectrometry Using a Linear Ion Trap. *J. Am. Soc. Mass Spectrom.* **2005**, *16* (9), 1498–1509, DOI: 10.1016/j.jasms.2005.04.017.
