# Supplementary material for: Novel Ambient Oxidation Trends in Fingerprint Aging Discovered by Kendrick Mass Defect Analysis
Source: ACS Cent Sci. 2022 Sep 21;8(9):1328–35. doi: 10.1021/acscentsci.2c00408 (PMC9523776; doi:10.1021/acscentsci.2c00408)
Supplement: Supplementary file 3 — oc2c00408_si_003.pdf [file oc2c00408_si_003.pdf]

oc-2022-004084.R1

Name: Peer Review Information for "Novel Ambient Oxidation Trends in Fingerprint Aging Discovered by Kendrick Mass Defect Analysis"

First Round of Reviewer Comments

Reviewer: 1

Comments to the Author

Comments

Paulson and Lee demonstrate in this work that Kendrick mass defect plot is good analysis and visualization tool for studying the aging products of fingerprints. By doing so they made two important discoveries: the epoxidation products of lipids and the medium chain fatty acids resulting from ozonolysis at the double bond position. They propose that the latter products have a potential to be used as chemical metrics for monitoring the aging process. Overall, the manuscript is well written, and the conclusions are supported by the data. The authors may wish to consider the following suggestions for further improvement of the manuscript.

1. Could the suspected epoxides be a combination of OH group and one increased degree of unsaturation of the original lipids? They share the same molecular formular. Performing MS/MS can provide evidence on the identity of epoxides.

2. The authors used an on-surface Paternò-Büchi (PB) reaction and tandem mass spectrometry to determine that the n-10 of C16:1 is one major double bond location in TG 48:2. It has been shown in several publications that MS/MS of the epoxides of lipids can provide information of the double bond location. The authors are suggested to acquire such data and compare the results with that acquired from the PB-MS/MS.

3. Because the overall goal of the research is to develop time-since-deposition models for fingerprints, the authors are suggested to demonstrate this point by using the newly discovered medium chain fatty acids.

4. Typo: Page 4, left column, line 1. Change “single oxygen” to “singlet oxygen”.

Reviewer: 2

Comments to the Author

Manuscript ID oc-2022-004084

Comments:

The article entitled “Novel Ambient Oxidation Trends in Fingerprint Aging Discovered by Kendrick Mass Defect Analysis” written by Paulson and Lee deals with the use of high-resolution mass spectrometry imaging combined with Kendrick mass defect analysis to detect and identify fingerprint lipids and their oxidation products. The Kendrick analysis provides an additional dimension to a highresolution mass spectrum (basically a one-dimensional graph, m/z and abundance) and produces a two-dimensional map which sorts / groups / disperses ion series in visual patterns and alignments. It helps the analyst to visualize congested mass spectra and assign series efficiently. In addition to the classification of oxidation products found in a 7-day aged fingerprint vs a fresh fingerprint based on a list of expected oxidation products generated in-silico, this analytical workflow led the Authors to report two main findings about the degradation processes of fingerprint lipids, namely an ambient epoxidation by singlet oxygen and a clear increase of fatty acid 10:0 over time as compared to the other FA congeners. Monitoring the abundance of these epoxides and FA 10:0 may provide a method to estimate the elapsed time since deposition of a fingerprint, of utmost importance in forensics.

The article is well-written and pleasant to read, and the findings convincing with elements of justification for the mechanisms leading to the epoxidation and the FA 10:0 abundance.

As a **major comment** justifying the recommendation as “major revision”, the KMD analysis is used in the present article to *display* the complex mass spectra and the IDs of series in figures. But the peak assignment is done by simulating expected compositions of oxidation products and screening the mass spectrum for hits, for which the KMD plot is of no use. I wonder if slightly more advanced Kendrick analysis would help the Authors actually assigning series from the KMD plots themselves without the need for the target list.

- For example, using *remainders* instead of NKM (or m/z, vide infra) would reduce the complexity of the plot by turning a horizontal alignment of point (= a series of congeners) into a single point. A remainder is calculated as follows with m(CH2)=14.01565 and m/z the mass-to-charge ratio of the ion of interest:

remainder = m(CH2) * (m/z / m(CH2) - Floor(m/z / m(CH2)))

All ions of a series have the same KMD and the same remainder, so a series is reduced to a point. The bimodal variation of abundance used to describe the expoxidized series will be lost but the overall ID of the oxidation products may be simplified. Can the Authors give it a try and compare the resulting plots ?

- The way the Authors computed their KMDs is a *static* normalization which produces only one plot per normalization moiety, here CH2 (KMD = m/z * round(m(CH2))/m(CH2)). Instead, they can try using a *dynamic* normalization computed as follows:

KMD=m/z * x/m(CH2), with 2/3*m(CH2) < x < 2*m(CH2)

By varying the value of x, the Authors can generate many different KMD plots with the same horizontal alignments but different vertical spread of points, leading to different separation of the series, some may overlap more but some may be better separated, facilitating the ID.

- Would the use of “O” instead of “CH2” reveal the number of oxidation / epoxidation reactions via the number of horizontally aligned congeners ? Or any other well-chosen normalization moiety beyond CH2 ?

Once more, I would like to see if the KMD plot can be used to *assign* peaks or understand the peak compositional relationships (+/-O, +/- other moieties), instead of helping *showing* the series once they have been assigned as it is done in the article.

I tried unsuccessfully to contact the Editor about the possibility for the Authors to share their raw data (e.g. the peak lists used for Figures 1 and 2, Figures S1 and S3 and even S8). I would be glad to give a try of these additional Kendrick processing techniques listed above in a second round of review.

A few **minor comments** too:

- the disodiated form of a carboxylic acid would be [M-H+2Na]+. In Supporting Information Fig S3, should we read $C_cH_{2c-Z}O_2Na$ and $C_cH_{2c-Z-1}O_2Na$? (same for the other degrees of oxidation)

- Since a target list has been generated, would it be possible to go one step further in the preprocessing and deconvoluting the data, e.g. merge the sodiated, disodiated and potassiated adducts into a single neutral component ? That would greatly reduce the complexity of the KMD map and the color / symbol coding, avoiding redundancy with FA or TG being observed twice as sodiated and disodiated congeners or the redundancy of potassium adducts.

- Since the Authors have recorded images of the fingerprints, is there any spatial variation for the oxidation products in terms of abundance and degree of oxidation ? (e.g. center vs edges of the fingerprint)

- About the difference between the mass spectra of the fresh and aged fingerprints (Fig 1), how would a simple subtraction look like ? Would the KMD plot of the subtracted spectrum (aged – fresh) be a bit simpler and better reveal the oxidation products ?

- About the KMD plot: there is no real need to plot KMD vs nominal Kendrick mass (NKM), KMD vs m/z works perfectly and it has the great advantage to retain the accurate mass in the graph.

- I strongly recommend using bubble plots instead of scatter plots for the KMD plot to retain the important information about point abundances via the size of the disk (it can be done by color coding but it would become difficult to ID series). A linear or logarithmic scale can be used for the disk sizes if peaks of interest are small, and that would prevent from using a "3D" plot (fig 5) to show the bimodal variation of peak abundance.

- In Scheme 1: do R1 and R2 become Rx and Ry on purpose ?

Reviewer: 3

Comments to the Author

The manuscript of Paulson and Lee describes a versatile methodology to shed light on lipids‘ oxidation during fingerprint aging. By using cutting edge mass spectrometry-based lipid analysis complemented by smart data visualization tools, the authors were able to decipher molecular mechanisms taking place

during fingerprint aging. This may pave the way for potential age dating of fingerprints. The original study is well designed and presented. Therefore, I suggest acceptance in ACS Central Science after minor revisions as listed below.

1. Highly complex mass spectrometric data is presented. Several lipids are contained which differ only in the number of double bonds. These data require consideration of ions with near-identical m/z values (i.e. isobaric masses), which comprises among others the Type-II isotopic overlap. This overlap occurs in series of lipid species differing only by number of double bonds mainly because of the natural abundance of 13C-atoms. High-resolution mass spectrometry, is capable of resolving Type-II overlap depending on mass resolving power (cf. J. Lipid Res. (2021) 62 100050; https://doi.org/10.1016/j.jlr.2021.100050).

Please check the data for such Type-II-isotopic overlap and address this issue in the manuscript.

2. Figure 2b: The assigned DBE may – at first glance – be misleading and refered to the number of double bonds. I suggest to add a short note to the figure caption that saturated TGs have DBE of 3 from three carboxylic groups.

Editorial:

Abstract: should it read "sparse ozone condition"?

Author's Response to Peer Review Comments:

Dear Editor,

We revised the manuscript to accord with your and the reviewers' comments as attached in the summary of the comment-by-comment response. We tried to make the main text as concise as possible, added most responses to the supporting information, and further moved some text to the supporting information, but still it is over by ~1000 characters (including space and references) which was inevitable to address the reviewers' comments. We hope it is acceptable. Thank you so much for all your help in handling this manuscript and let me know if we need anything else to make this manuscript acceptable for the publication.

Sincerely,

Young-Jin Lee

Summary of Revisions and Responses

We are grateful for insightful comments from the reviewers to enhance the quality of this manuscript. We tried our best to accord with reviewers' comments. We believe the revised manuscript is a much higher quality and hope it is acceptable for the publication. Our comment-by-comment response is shown below in red with “>>” following each of the reviewers’ comment. We also made minor changes summarized below.

Notable minor changes not requested:

1) After more literature search, we decided to give some clarification in epoxidation mechanism. Edited in text explanation of epoxide origin (pg 4, 4th paragraph)

   “The mechanism proposed by Weiny et al. involves a peroxyl radical intermediate, the same as Zhou et al.’s, but the epoxides are suggested as the final products.[26,27] Therefore, singlet oxygen is a promising source for peroxyl radical intermediates in ambient conditions to explain the subsequent epoxide formation, though other autooxidation mechanisms may also lead to the peroxyl radical. There is contradictory literature evidence for the initial hydrogen abstraction. Specifically, Wu et al. suggested that minimal epoxidation occurs for lipid monolayers or bulk systems containing exclusively monounsaturated aliphatic chains under heat.[28] Thus, further investigation is necessary for the origin of peroxyl radical intermediate formation in fingerprint lipids.”

2) As detailed in the response to the reviewers, we added extensive discussion and more data analysis. Due to the character limit, we had to move most of them to the supporting information. To save additional characters, we also had to move some more contents to the supporting information including most of the methods.
3) Other minor changes are made throughout the manuscript in some statements, figures, tables, and scheme 2 to improve clarification.

Response to Editor's Notes:

Key points that seem important to me include the following:
- further direct MS/MS including a comparison of epoxide MS/MS where appropriate (ref 1)
- the interesting suggestion to adopt a remainder-based approach (ref 2)
- dynamic normalization (ref 2)
- Type-II-isotopic overlap (ref 3)

>> They are addressed as summarized in each response to the reviewers.

Please also provide the requested raw data via an archive (e.g. Zenodo).

>>As the requested file size is not too big and ACS accepts .zip file, it is now directly attached as Supporting Information for Publication so that the readers and reviewers can have a direct access without having to go through the third-party data repository.

Response to Reviewer 1

1) Could the suspected epoxides be a combination of OH group and one increased degree of unsaturation of the original lipids? They share the same molecular formular. Performing MS/MS can provide evidence on the identity of epoxides.

>>We added a comment that we cannot avoid the possibility in page 4, right column, 1st paragraph:

"... However, we cannot avoid the possibility that some isomeric species may also contribute to the features assigned as epoxides, such as the termination of structure D (**Scheme 2**) with another abstracted hydrogen to form an enol"

But the stability of the fully saturated epoxides (**Figure 4**) suggests that the carbon-carbon double bond is likely no longer present after the oxidative process. A related statement is added in page 5: 3rd paragraph.

"The stability of the homologous series suggests that the carbon-carbon double bond is likely no longer present, consistent with fully saturated epoxides instead of enol."

MS/MS of fingerprint lipids:

An attempt at ESI-MS/MS experiments on the epoxides of TGs directly from fingerprint extracts did not produce sufficient signals, likely due to the low signal of the epoxide peak, especially compared to co-isolated (minimum isolation window of 0.4 Da) species (e.g., TG(E)48:0 is only 36.4 mDa apart from TG49:0). A fragment consistent with the neutral loss of [FA+NH_3] from the TG is observed when doing ammonium acetate doped ESI, leaving the extra oxygen on the structure, though this isn't all that informative.

MS/MS was also attempted on FA(E)16:0 in negative mode as $[FA16:0(E)-H]^-$ from fingerprint extracts. Signal of FA(E)16:0 was still poor, but fragments consistent with an epoxide on the □-10 position are present above a S/N of 3 (See **Figure S11** in the next response about PB rxn comparison).

MS/MS of aged TG standard:

As MS/MS analysis of the FP extract was challenging, MS/MS was performed on an aged TG standard as an alternative. Thin films (~4nm) of a mixture of standards, 1,3-dipalmitoyl-2-oleoyl glycerol (TG50:1) and glycerol tripalmitate (TG48:0), were prepared on glass slides. Samples were aged for 24 hours in ambient lab conditions. In the ESI-MS spectrum of the extract, mass peaks were observed with one and three oxygens added to the TG 50:1. The MS/MS fragmentation for the proposed epoxide is consistent with an epoxide fragmentation (see the figure below, note the standard has an □-9 double bond position).The fragment peaks indicative of an epoxide are the loss of [C_9H_{18}] and [$C_9H_{18}O$] pair (dotted and dashed arrow) in addition to the neutral loss of [FA16:0+NH_3] from the TG(E)50:1. **Figure S12** is included in Supplementary Information:

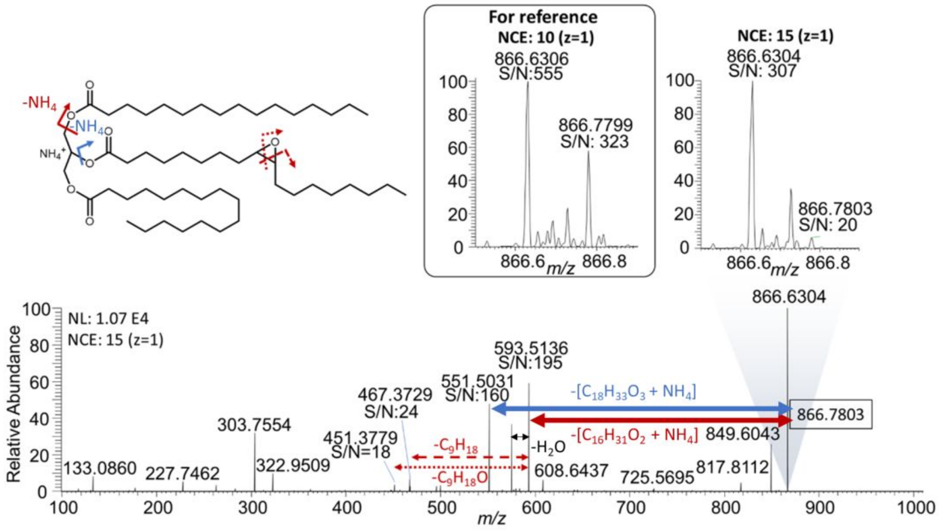

Figure S12. Direct infusion ESI-MS/MS of TG(E)50:0, $[M+NH_4]^+$, from an extracted thin film (~ 4 nm) of TG standard mixture (1:1, TG 50:1: TG48:0) aged in ambient laboratory conditions for one day. The thin film was extracted with MeOH:$CHCl_3$ (1:1) doped with 5 mM of ammonium acetate to promote ionization. Arrows in the structure indicate fragmentation channels corresponding to the major fragments in the MS/MS spectrum. Dotted and dashed arrows are characteristic of epoxide fragmentation occurring in parallel to FA16:0 acyl chain fragmentation. A normalized collision energy (NCE) of 15 is used for the theoretical *m/z* 866.7807 with the isolation window of ±0.2 Da. There is a co-isolated interference ion at *m/z* 866.6304 but it does not appear to undergo significant fragmentation compared to *m/z* 866.7807 as shown in the inset zoomed-in spectra for the precursor at NCE of 10 and 15.

Commented [LYJ[1]: too long. need to cutdown to essentials as the details is in the method section.

Discussion in regard to the MS/MS spectrum is added in the text, page 4, right column, 1st paragraph:

"... ESI-MS/MS of TG(E) from the fingerprint extracts was not successful due to poor signals, but MS/MS of FA(E) 16:0 from the same extract suggests the presence of epoxides (**Figure S11**). Additionally, ESI-MS/MS on the extract of aged TG standards, a mixture of TG 48:0 and TG 50:1, have fragmentation patterns consistent with epoxides (**Figure S12**)."

2) The authors used an on-surface Paternò-Büchi (PB) reaction and tandem mass spectrometry to determine that the n-10 of C16:1 is one major double bond location in TG 48:2. It has been shown in several publications that MS/MS of the epoxides of lipids can provide information of the double bond location. The authors are suggested to acquire such data and compare the results with that acquired from the PB-MS/MS.

>>As mentioned above, ESI-MS/MS experiments on the TG epoxides directly from fingerprint extracts were not successful due to low signals. However, the analysis of FA(E)16:0 in negative mode from the same fingerprint extracts produced fragments consistent with an epoxide on the □-10 position, although low intensity slightly above a S/N of 3. It contrasts with PB reaction that produces significantly high precursor ion signals for MS/MS. Another benefit of the PB reaction on TGs is that the mass shift of the

derivatized compound is significant enough to move the derivatized peak out of the congested spectral region to allow for isolation and subsequent fragmentation.

Discussion in regard to the double bond position is added in the text, page 6, 1st paragraph:
"... ESI-MS/MS of the FA(E) 16:0 is consistent with the prevalence of sapienic acid in the fingerprints (**Figure S11**)."

Commented [a2]: Double check before submission

Figure S11 is included in the supplementary information.

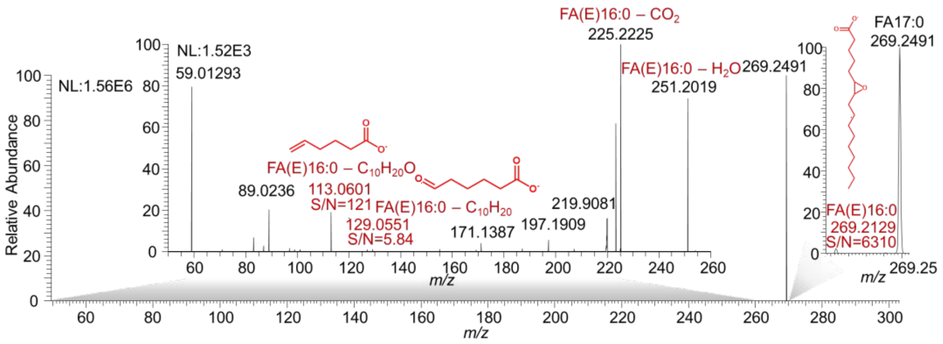

Figure S11. Direct infusion ESI-MS/MS of FA(E)16:0, $[M-H]^-$, from extracted fresh fingerprints. FA17:0, *m/z* 269.2491, is a co-isolated ion that is not expected to undergo significant fragmentation. A normalized collision energy of 30 is used. The mass error is less than ± 7 ppm for all assignments. The larger mass error tolerance is due to the poor calibration of *m/z* values below the *m/z* of dodecyl sulfate (*m/z* 265.1479), the lowest value used for negative mode calibration.

3. Because the overall goal of the research is to develop time-since-deposition models for fingerprints, the authors are suggested to demonstrate this point by using the newly discovered medium chain fatty acids.

>>We have included the following figure in the supplementary information to show the promise of using various spectral features such as medium chain fatty acid fragments for accurate time since deposition estimations. We intend to develop such a model in a systematic large-scale study in the future work with controlled kinetics experiments or machine learning approaches.

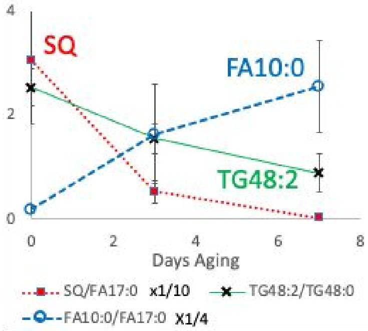

Figure S18. Temporal trends of promising targets for fingerprint time-since-deposition models.

We added statements in the text page 6, 3rd paragraph:

“A robust model for time since deposition should have multiple spectral features to describe different periods of the aging. In such a model squalene oxidation may best describe early aging (<3 days), TG degradation captures mid-range aging (2-7 days), and the accumulation of FA 10:0 has the potential to verify and extend the 7-day aging window (**Figure S18**). Future work will focus on kinetics and machine learning based approaches to generate these models with larger data sets.”

4. Typo: Page 4, left column, line 1. Change “single oxygen” to “singlet oxygen”.

>>Changed in text.

Response to Reviewer 2

As a major comment justifying the recommendation as “major revision”, the KMD analysis is used in the present article to display the complex mass spectra and the IDs of series in figures. But the peak assignment is done by simulating expected compositions of oxidation products and screening the mass spectrum for hits, for which the KMD plot is of no use. I wonder if slightly more advanced Kendrick analysis would help the Authors actually assigning series from the KMD plots themselves without the need for the target list.

>>A major goal of this work is to demonstrate that the majority of aged compounds can be explained by ambient ozonolysis and this justifies the approach we took with the target list. The suggested plots are attempted below and we found them very useful, but we are ultimately limited by spectral resolving power and mass accuracy for the assignment. Given that we are already over the character count limit and we are trying to keep the scope of the analysis readily accessible to the broad audience, we incorporated the discussion of such plots in the supporting information and only briefly mentioned in the text, Page 3, right column, 1st paragraph:

"... Investigating unannotated features is aided with using different KMD plot normalization techniques which are demonstrated and described in the supporting information (Supporting discussion, **Figure S7-S9**)."

The KMD plots presented in this work are still very useful for parsing out clusters, especially those corresponding to lower intensity spectral features, that were not explained by our previous understanding of the ionization and/or oxidation processes that were occurring with a targeted look at triacylglycerols. For example, we did not anticipate the epoxides and would not have found them without simplifying the spectral trends with the KMD approach, noticing that they align with the aldehyde series from ozonolysis (using the KMD plots to aid assignment). Also, we had not previously investigated [FA+2Na-H]+ adducts and had assumed that we simply had poor reproducibility of $[FA+Na]^+$ adducts. Through the clustering that results from KMD it was much easier to streamline these assignments by focusing on unidentified/unexplained trends, identifying the elemental composition, establishing the origin of the compound series, and then adding it to the target list. Subtle differences within the intensity profiles were also easy to interpret with the added third axis of intensity within the plot, which is more challenging to do with the bubble representation due to potential overlap of the bubbled features.

For example, using remainders instead of NKM (or m/z, vide infra) would reduce the complexity of the plot by turning a horizontal alignment of point (= a series of congeners) into a single point. A remainder is calculated as follows with m(CH2)=14.01565 and m/z the mass-to-charge ratio of the ion of interest:
remainder = m(CH2) * (m/z / m(CH2) - Floor (m/z / m(CH2)))

All ions of a series have the same KMD and the same remainder, so a series is reduced to a point. The bimodal variation of abundance used to describe the expoxidized series will be lost but the overall ID of the oxidation products may be simplified. Can the Authors give it a try and compare the resulting plots?

>>In contrast to the reductionist approach suggested with the remainder plots, we believe plotting against *m/z* (changed from NKM based on the reviewer suggestion) is more useful in this case as some overlapping chemical class but different series (e.g., DG(E) vs TG) can be only distinguished in *m/z* dimension. However, we did give this approach a try, see below.

Remainder Plots:

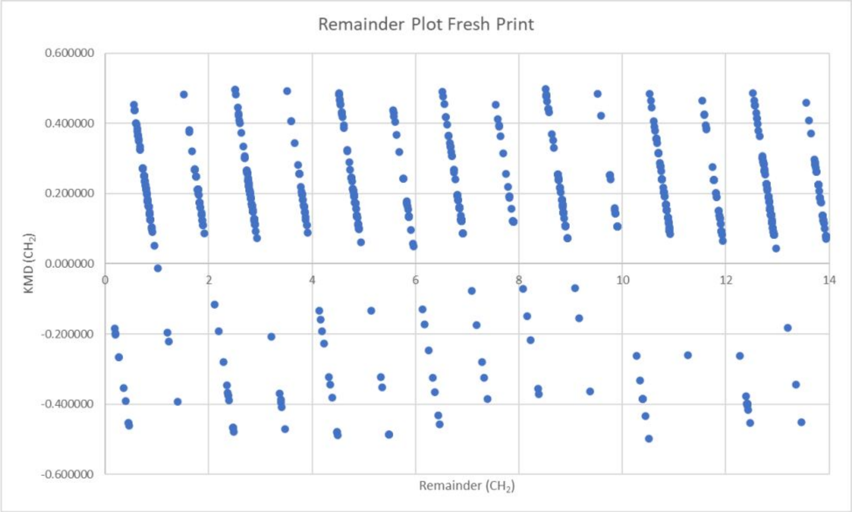

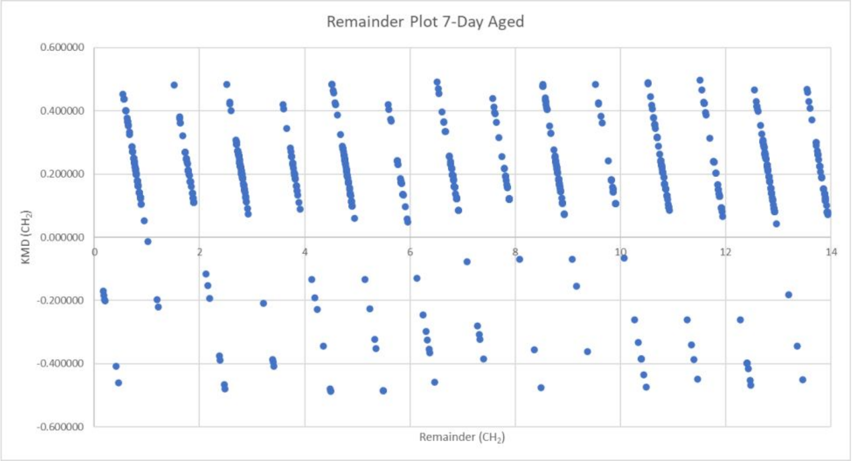

The way the Authors computed their KMDs is a static normalization which produces only one plot per normalization moiety, here CH2 (KMD = m/z * round(m(CH2))/m(CH2)).
Instead, they can try using a dynamic normalization computed as follows:
KMD=m/z * x/m(CH2), with 2/3*m(CH2) < x < 2*m(CH2)
By varying the value of x, the Authors can generate many different KMD plots with the same horizontal alignments but different vertical spread of points, leading to different separation of the series, some may overlap more but some may be better separated, facilitating the ID.

>>We see the benefit of these plots for the original discovery of trends and have added the dynamic normalization plot using x=9 to the supplementary information as well as an explanation of the plot generation (note most of the unassigned peaks are ^{13}C peaks, which are annotated in the normalized plot). A new supporting discussion section is dedicated with the following statements, on page 3 of the supporting information describing **Figures S7**:

"**Describing undefined plot features.** There are series in the KMD plots that were not annotated with the list described and not obviously explained by ozonolysis. Further finding unassigned trends can be aided by dynamic normalization, which is similar to fractional base units described by Fouquet *et al*.[3] **Figure S13** is an example of this normalization where KM is equivalent to the product of the *m/z* and x, user specified number, divided by the IUPAC exact mass of the Kendrick base unit. This method changes the vertical dispersion of different homologous series, while maintaining the horizontal alignment within a homologous series. This can allow for the interpretation of closely aligned series observed when using static normalization alone. Using the dynamic KMD plot with x = 9, ^{13}C isotope series could be successfully separated and annotated in **Figure S7**. "

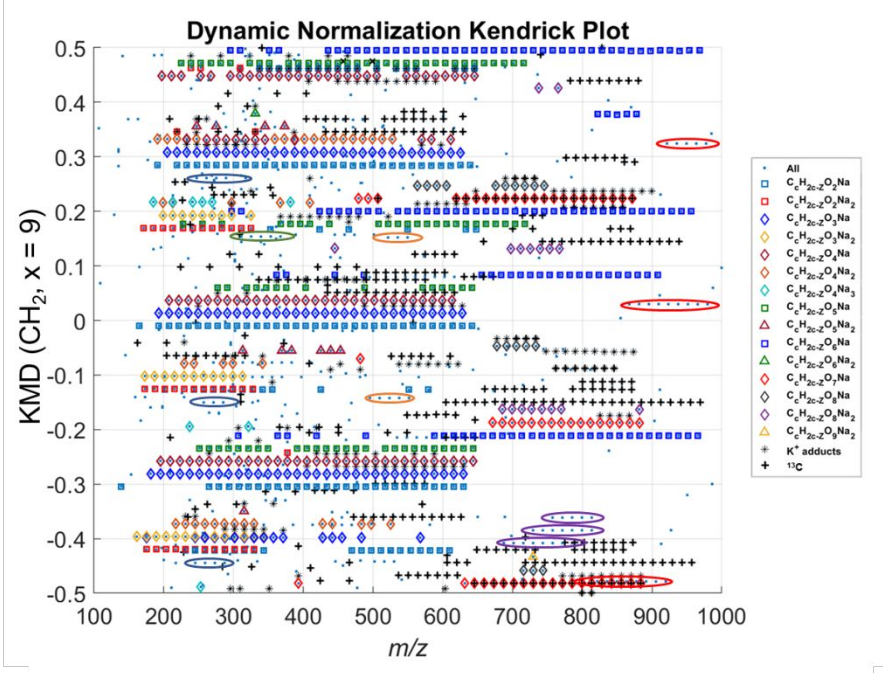

Figure S7. Dynamic normalization KMD plot of the 7-day aged fingerprint with x=9.

Foot notes for Figure S7:

1. ^{13}C isotope contributions, up to three ^{13}C atoms for all searched *m/z* values, were included and annotated in this plot.

2. The dynamic normalization uses a different calculation for the KMD:

KMD = *m/z* * x/(14.01565)

Instead of x=14, the user inputs other values in order to manipulate the vertical dispersion of different homologous series while retaining the horizontal alignment within a homologous series. This allows visualizing homologous series that are nearly overlapped with static, x=14, normalization.

3. Note that a majority of the plot features are defined. Most of the unassigned features are well separated in the x=14 plot, and are aliased into the KMD range of the defined lipids when dynamic normalization is used (features circled red). However, this plot serves to check to ensure homologous series are not overlooked. The relationships of heteroatom compositions of unassigned features can be aided with using a different KMD base, as described in Figure S11 and S12.

4. The features circled in blue are tentatively assigned as sodiated adducts of unsaturated aldehydes, $C_cH_{2c-Z}ONa$ (Z_{min} = 2), and those circled in green as sodiated adducts of monoacylglycerols, $C_cH_{2c-Z}O_4Na$ (Z_{min} = 0), where assignments were assisted by using a KMD base of O in Figure S11.

5. The features circles in purple are an unassigned heteroatom classes that differ by CO, as seen in Figure S12.

6. The features circles in orange are an unassigned heteroatom class with exact mass consistent with $C_cH_{2c-Z}O_4$ (Z_{min} = 2), as protonated adducts.

7. Note the misassignment of the $C_cH_{2c-Z}O_7Na$ features as the ^{13}C isotope peaks. This is an obvious misassignment considering the lack of monoisotopic peaks.

Further, X= 28 and 16 are also attempted as shown below but were not as useful to provide additional information.

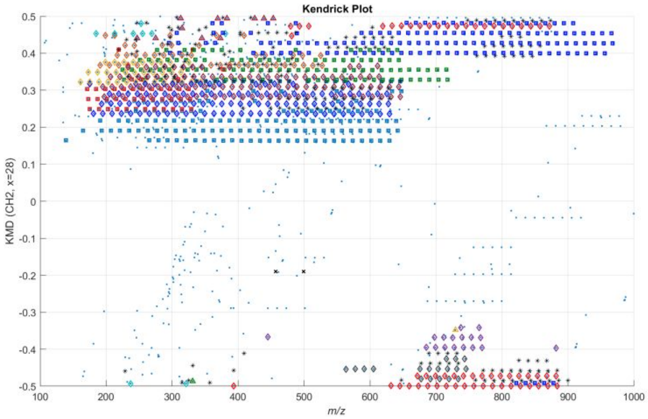

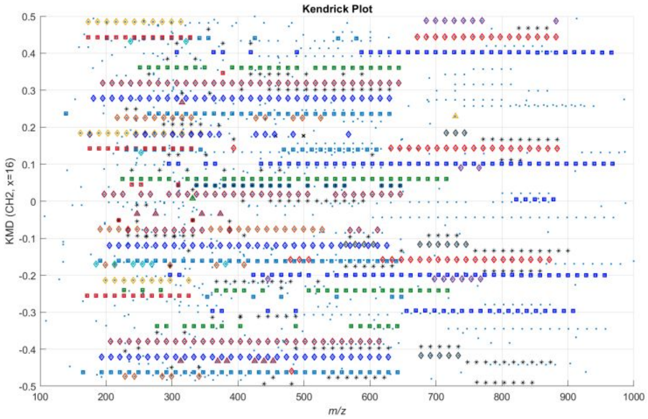

- Would the use of “O” instead of “CH2” reveal the number of oxidation/epoxidation reactions via the number of horizontally aligned congeners? Or any other well-chosen normalization moiety beyond CH2? Once more, I would like to see if the KMD plot can be used to assign peaks or understand the peak compositional relationships (+/-O, +/- other moieties), instead of helping showing the series once they have been assigned as it is done in the article.

>>Such analysis is performed and **Figure S8** and **S9** are included in Supplementary Information.

The following discussion is added in the supporting discussion section on pg 3 of the supporting information.

“**…** After accounting for ^{13}C isotopes, there are still some undefined series that need further investigation, some of which were investigated by the plots generated with different KMD base units. An example is using atomic oxygen, $^{16}O_1$ as a KMD base unit (**Figure S8**). Though the information is also in the CH_2 KMD plots, differences in oxygen content can easily be observed in this plot. We highlight two heteroatom

classes that are more clearly observed in these plots. Including a series with high oxygen content in the fresh prints, which could be exogenous compounds (**Figure S8c**), and a heteroatom class of $C_cH_{2c-Z}ONa$ in the low mass range (*m/z* 250-300) which are tentatively assigned as unsaturated aldehydes (**Figure S8d**). Aldehyde species are expected to result from ozonolysis but the resulting peaks are not in the expected mass range. Instead, they are comprised of 16 to 18 carbons and are present in the both the aged and fresh fingerprints. A KMD plot using $^{12}C_1{}^{16}O_1$ as a base unit helps detect a cluster of features present primarily in the 7-day aged plot, but these features are not likely to be associated with aging as they are near the S/N>30 cutoff and are also present in the fresh spectrum at a similar S/N (**Figure S9**). "

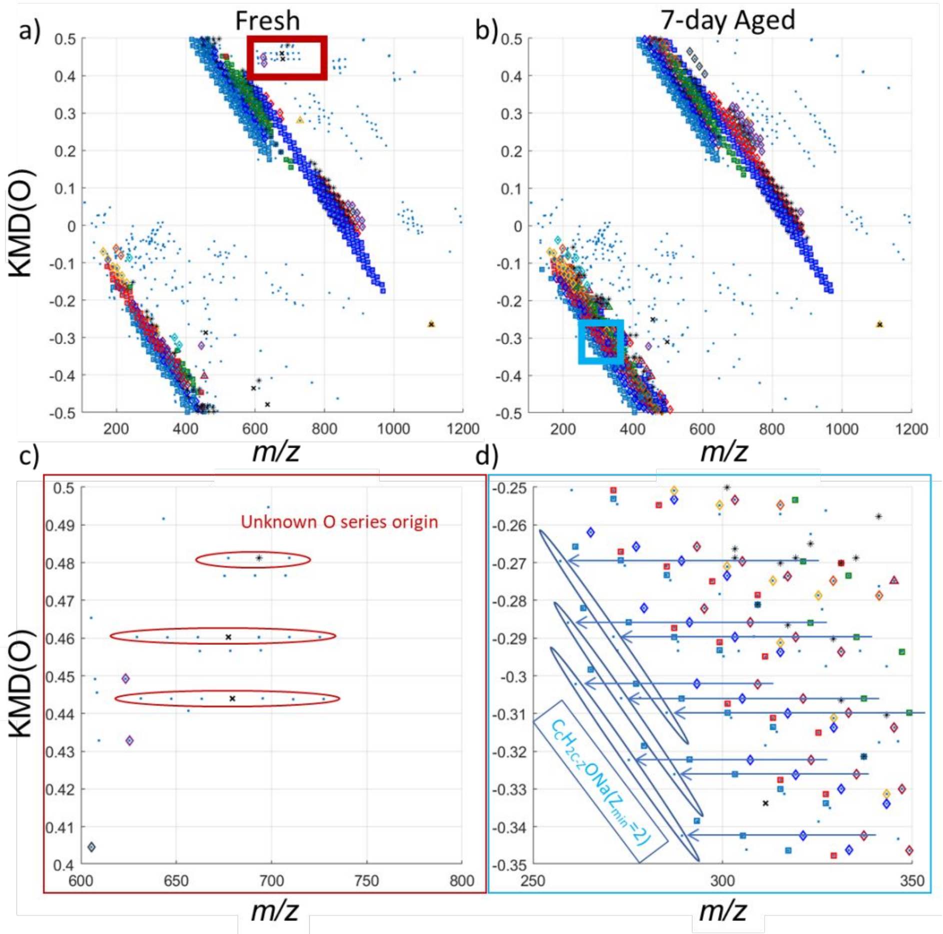

Figure S8. KMD plots using ^{16}O as a Kendrick mass base unit for the **(a)** fresh and **(b)** 7-day aged fingerprints. Horizontal alignments helped assign the elemental compositions of **(c)** a group of plot features differing by oxygen content present in the fresh print that have an unknown origin, and **(d)** a heteroatom class tentatively assigned as unsaturated aldehydes that was previously unassigned.

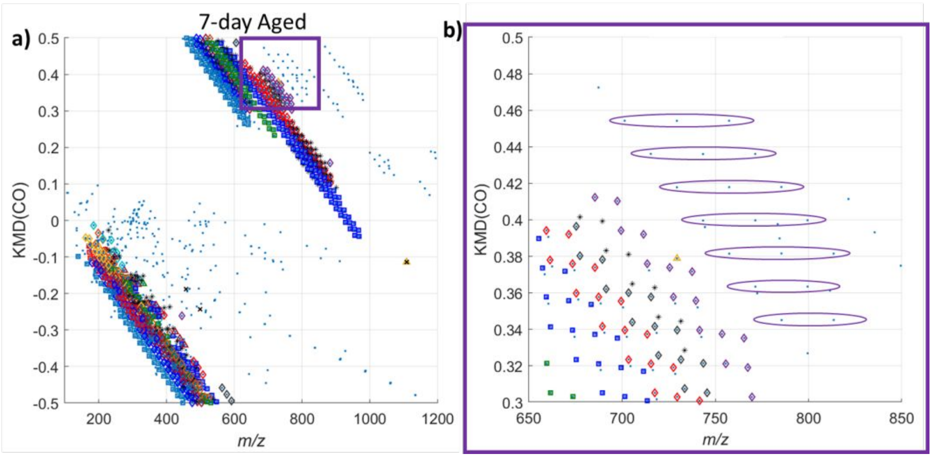

Figure S9. KMD plots using $^{12}C_1{}^{16}O_1$ as a Kendrick mass base unit for the (**a**) 7-day aged fingerprints. Horizontal alignments helped visualize (**b**) a group of unassigned plot features differing by CO oxygen content present in the 7-day aged, though the origin is unknown.

I tried unsuccessfully to contact the Editor about the possibility for the Authors to share their raw data (e.g. the peak lists used for Figures 1 and 2, Figures S1 and S3 and even S8). I would be glad to give a try of these additional Kendrick processing techniques listed above in a second round of review.

>>We did send the data following the request from the editorial office. We are not sure where it was lost. It is now attached as Supporting Information as a .zip file.

A few minor comments too:
- the disodiated form of a carboxylic acid would be [M-H+2Na]+. In Supporting Information Fig S3, should we read CcH2c-ZO2Na and CcH2c-Z-1O2Na ? (same for the other degrees of oxidation)

>>The minimum Z values for each class is observed in the table S2. For each increase in DBE, Z increases by 2. Please note that the Z is an odd value for the disodiated fatty acid.

To clarify the following footnote was added to the footnote for Table S2:

"3. Z is an odd value for disodiated heteroatom class and even otherwise. It increases by 2 for each DBE increase."

- Since a target list has been generated, would it be possible to go one step further in the pre-processing and deconvoluting the data, e.g. merge the sodiated, disodiated and potassiated adducts into a single neutral component? That would greatly reduce the complexity of the KMD map and the color / symbol coding, avoiding redundancy with FA or TG being observed twice as sodiated and disodiated congeners or the redundancy of potassium adducts.

>>A major goal of this work is to explain the spectral complexity of the aged fingerprint, both due to aging and ionization, and therefore the original data was not preprocessed. Now that the plot features are identified, they can be merged for the use in aging models, if the analyst chooses to do so.

Since the Authors have recorded images of the fingerprints, is there any spatial variation for the oxidation products in terms of abundance and degree of oxidation? (e.g. center vs edges of the fingerprint)

>>This is an interesting question we may consider in the future, but we never acquire MS imaging of an entire fingerprint. It is a violation of our IRB protocol because a fingerprint image can be used to identify an individual. Additionally, it takes too much time to collect a dataset for the entire fingerprint. With the data collected for a small section of a fingerprint, there seems to be minimal heterogeneity within the same type of oxidation process, now added in **Figure S10**.

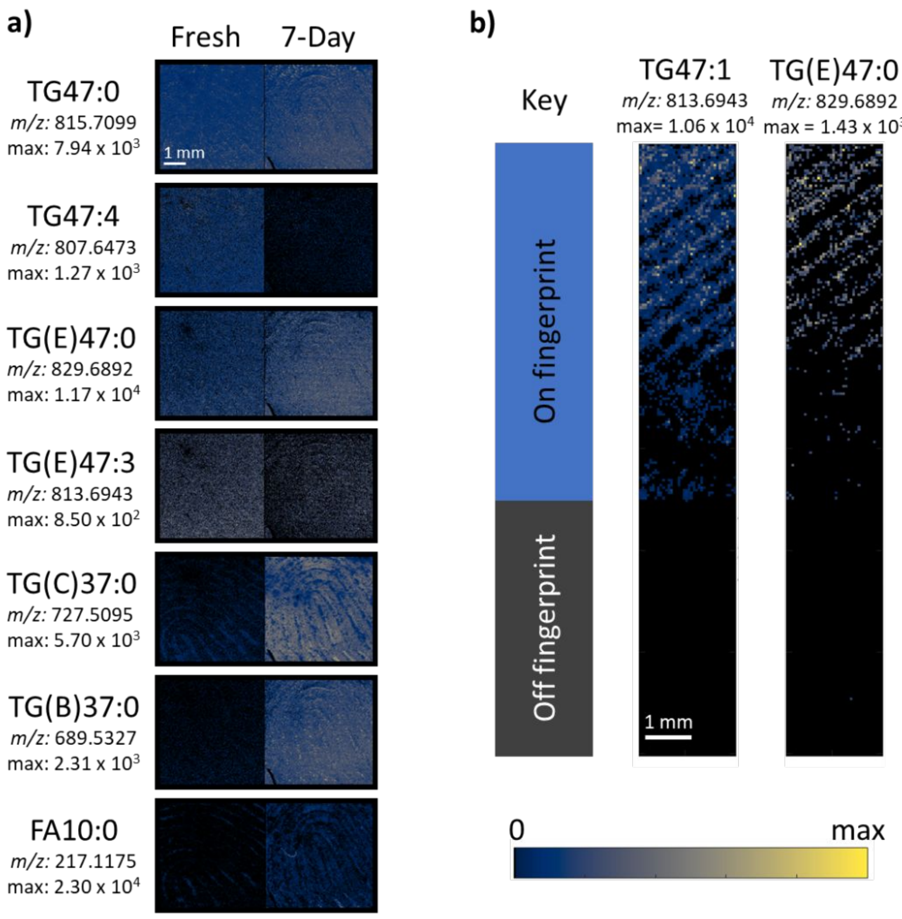

Figure S10. (a) Mass spectrometry images of a selection of *m/z* values (± 2 ppm) of interest for a section in the middle of a fresh and 7-day aged fingerprint (20 µm raster step). (b) Mass spectrometry images of TG47:1 and TG(E)47:0 at the edge of a fresh fingerprint (50 µm raster step). All adducts are $[M+Na]^+$ except TG(C)37:0, which is $[M-H+2Na]^+$.

- About the difference between the mass spectra of the fresh and aged fingerprints (Fig 1), how would a simple subtraction look like? Would the KMD plot of the subtracted spectrum (aged – fresh) be a bit simpler and better reveal the oxidation products?

>>It is a good suggestion. Now Figures 1a (fresh) and 1b (aged) are replaced with the subtracted spectrum. This allows for the easy identification of 1) significantly decreasing peaks (e.g., squalene) and 2) new peaks in aged spectrum (e.g., ozonolysis products). In order to keep the explanation simple, however, we opted to keep the unsubtracted original overlain KMD plot as Figure 1c. As shown below, the subtracted Spectrum KMD Plot does not provide any additional information compared to the original KMD plot (Not included in text):

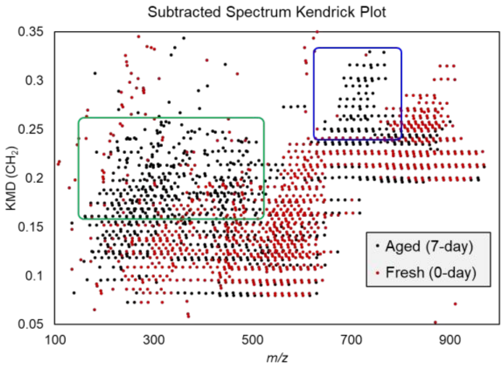

Due to the change in Figures 1a and 1b, significant change is made to the first two paragraphs of the Results and Discussion pg 2, 2nd and 3rd paragraph:

Main text:

“**Subtracted spectra and KMD plots for general spectral differences**. A high-resolution Orbitrap MS connected with a MALDI source is used in this study for the analysis of aged fingerprints in ambient lab conditions. **Figure 1** is the subtracted spectrum and the overlain KMD plots in the lipid KMD range (0.05-0.35) for the fresh (0-day) and 7-day aged fingerprints. To generate the subtracted spectrum, all spectral features were normalized to the summed signal of saturated TGs to account for differences in deposition. The original spectra (**Figure S1**), full overlain KMD plot (**Figure S2**), and the environmental conditions during the aging (**Figure S3**, **Table S1**) can be found in the supporting information. KMD bubble plots are also helpful as they retain the relative abundance information of the plot features (**Figure S4**).

Many noticeable differences can be found in the subtracted mass spectra. Most notable is the loss of SQ (*m/z* 433.3805) in the aged fingerprint (**Figure 1a**). This is consistent with previous findings of rapid decay of SQ by GC-MS and LC-MS.[22,23] Other differences, such as the negative peaks in *m/z* 700-900 and positive peaks in *m/z* 600-800 (**Figure 1b**), are related with the fingerprint aging and clearly distinguished in the KMD plot (**Figure 1c**). Thus, the spectral and plot differences can be used in concert in order to identify time-dependent *m/z* features and related series to understand the molecular details of the aging process over time. Previously, we proposed to use the degradation of TGs to monitor the time since deposition of fingerprints due to high ion abundance and multiple levels of unsaturation.[10] The ozonolysis products for TGs are readily observed as the new cluster of KMD plot features in the *m/z* range of ~550-750 (blue box). However, oxidative aging of WEs, DGs, FAs, and SQ can also be studied using MALDI-HRMS with KMD analysis (green box) as described in more detail later.”

Figure 1 included in main text:

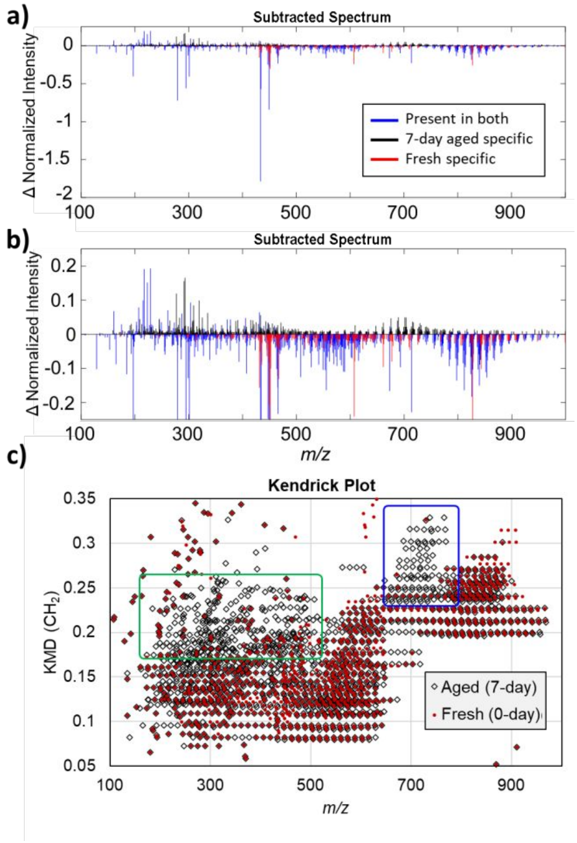

Figure 1. (a) Subtracted MALDI-MS spectrum, where the normalized spectrum of the fresh fingerprint was subtracted from the normalized 7-day fingerprint spectrum. **(b)** Zoom in along the y-axis of the subtracted spectrum. **(c)** Overlain KMD plot of the fresh (red circles) and 7-day old (black diamond) fingerprints for S/N>30.

- About the KMD plot: there is no real need to plot KMD vs nominal Kendrick mass (NKM), KMD vs m/z works perfectly and it has the great advantage to retain the accurate mass in the graph.

>>We agree. Figures, references to values, and plot explanations were changed to use *m/z* as x-axis instead of NKM.

- I strongly recommend using bubble plots instead of scatter plots for the KMD plot to retain the important information about point abundances via the size of the disk (it can be done by color coding but it would become difficult to ID series). A linear or logarithmic scale can be used for the disk sizes if peaks of interest are small, and that would prevent from using a "3D" plot (fig 5) to show the bimodal variation of peak abundance.

>>A bubble plot using a linear scale for fresh and 7 Day Aged has been added to the supplementary information for the lipid space of the KMD Plot. The third interactive intensity axis was beneficial for trend discovery, especially for subtle differences in signal intensity. We did not replace the main figure with the bubble plots, as it becomes too complicate with the annotation style, as the reviewer noted.

Added to main text on pg 2, 2nd paragraph: “KMD bubble plots are also helpful as they retain the relative abundance information of the plot features (**Figure S4**).”

Figure S4 is included in Supplementary Information:

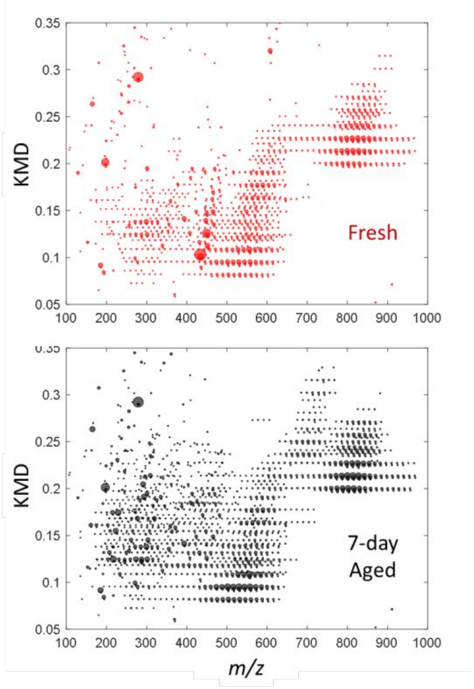

Figure S4. Kendrick mass defect bubble plots of the fresh (red) and 7-day old (black) fingerprints where bubble size is proportional to the relative abundance.

Fig 5a was updated to bubble plots of the saturated FAs in the main text:

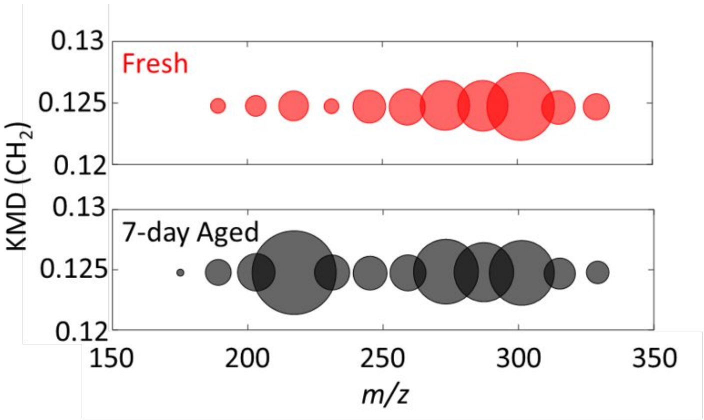

Figure 5. (a) KMD bubble plot for saturated fatty acids ($C_cH_{2c\text{-}Z}O_2Na_2$, DBE=1) in fresh and 7-day aged fingerprints.

- In Scheme 1: do R1 and R2 become Rx and Ry on purpose ?

>>Rx and Ry were changed in the figure to R_1 and R_2.

Response to Reviewer 3

1. Highly complex mass spectrometric data is presented. Several lipids are contained which differ only in the number of double bonds. These data require consideration of ions with near-identical m/z values (i.e. isobaric masses), which comprises among others the Type-II isotopic overlap. This overlap occurs in series of lipid species differing only by number of double bonds mainly because of the natural abundance of 13C-atoms. High-resolution mass spectrometry, is capable of resolving Type-II overlap depending on mass resolving power (cf. J. Lipid Res. (2021) 62 100050; https://doi.org/10.1016/j.jlr.2021.100050). Please check the data for such Type-II-isotopic overlap and address this issue in the manuscript.

>>We do have a sufficient revolving power to separate Type-II overlap for most ions and the following statement was added to the manuscript on pg 3, 2nd paragraph:

"The mass resolving power used is sufficient to resolve Type-II isotopic overlap ($\Delta m/z$ of 0.0089 between the second ^{13}C peak and one more saturation).[25] As we are approaching the limits of the resolving power, however, some ^{13}C peaks are unresolved when the relative ion signal is very low (**Figure S6**). Regardless, it has minimal impact on heteroatom class assignment based on the ± 2 ppm mass tolerance."

Some examples of unclear separation are included in Supplementary Information (**Figure S6**):

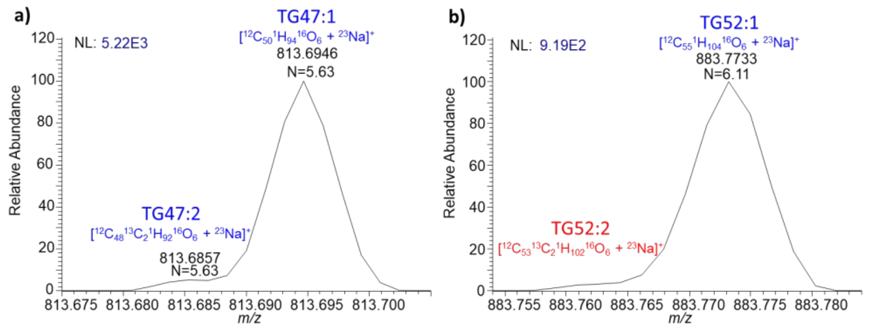

Figure S6. Type-II isotopic overlap within the 7-day aged fingerprint spectrum demonstrating some limitations of the instrumentation at a higher *m/z* range, especially with drastic signal differences.

2. Figure 2b: The assigned DBE may – at first glance – be misleading and refered to the number of double bonds. I suggest to add a short note to the figure caption that saturated TGs have DBE of 3 from three carboxylic groups.

>>The following statement was added to the figure caption:

“Note the DBE of saturated TGs is three due to the three esterified carboxyl groups.”

Editorial:
Abstract: should it read "sparse ozone condition"?

>>Changed in text

oc-2022-004084.R2

Name: Peer Review Information for "Novel Ambient Oxidation Trends in Fingerprint Aging Discovered by Kendrick Mass Defect Analysis"

Second Round of Reviewer Comments

Reviewer: 3

Comments to the Author

The authors addressed all my concerns adequately. Therefore, I suggest acceptance.

Reviewer: 1

Comments to the Author

The authors have adequately addressed the concerns with new data acquired and related discussions added. The quality of the revised manuscript has been improved. Therefore, I fully support publication of the manuscript in its current form.

Reviewer: 2

Comments to the Author

Dear Authors,

I do sincerely praise the quality of the second version of the manuscript, and the efforts put in the revision. All the comments of the Referees have been carefully considered, with additional comments, figures and procedures thoroughly explained in the draft or the supporting information.

I now strongly recommend the submitted draft to be published as is.

FYI, You will find a few figures attached, showing an example of "dynamic" Kendrick plot with "O" as the rescaling unit revealing oxidation lines, as well as an example of Kendrick plot using benzophenone as the rescaling unit to easily find the reaxtion produtcs of the Paternò–Büchi derivatization.

Kendrick plot using “O” as the rescaling unit and x=16
Blue points correspond to potential +/- n*O series (+/- 1mDa tolerance)

“Fresh_6”

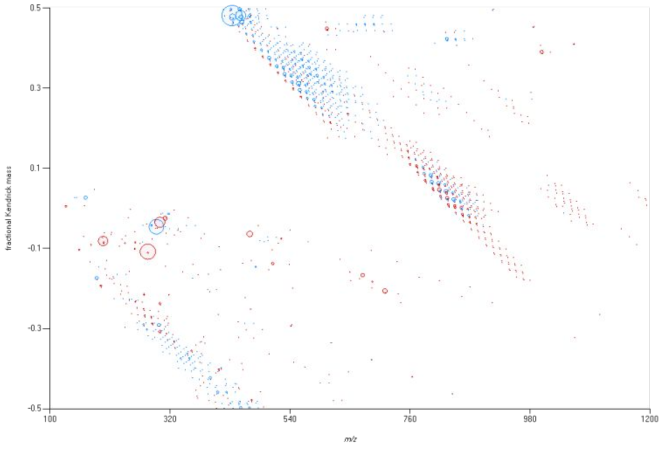

“7day_3”

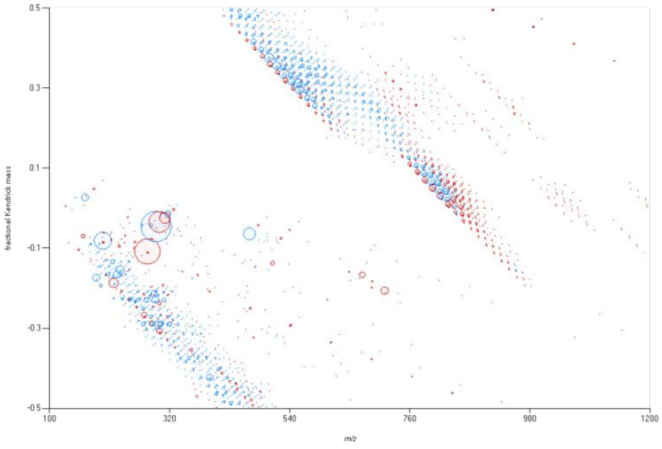

Kendrick plot for “7day_3”, using “O” as the rescaling unit and x=15

“Fresh_6”

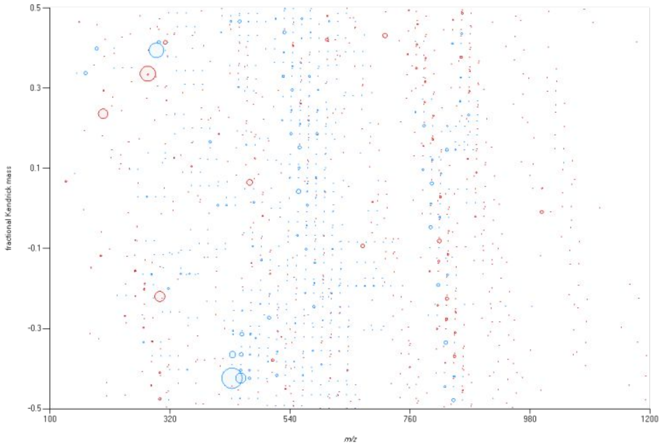

“7day_3”

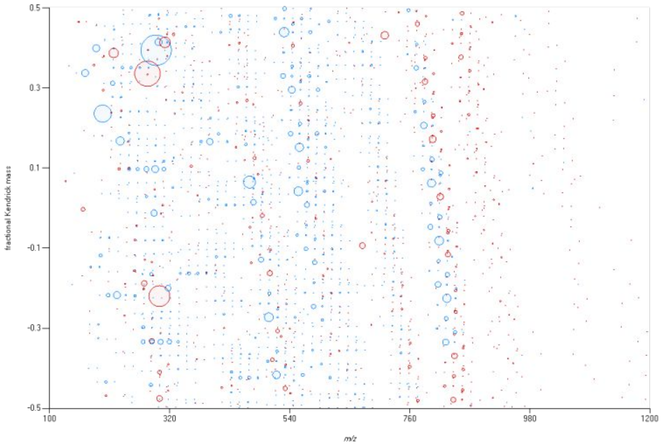

Alternative representation of the dataset using a Remainder plot for “7day_3”, using “O” as the rescaling unit and x=15
+/- n*O series are revealed via the superimposition of points at the same remainder and Kendrick mass defect values

“Fresh_6”

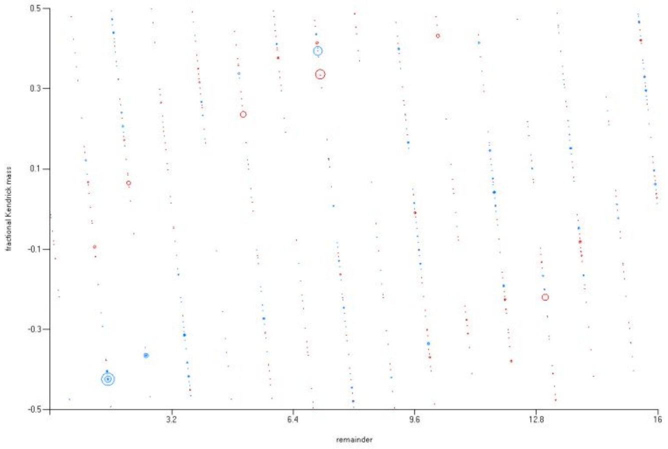

“7day_3”

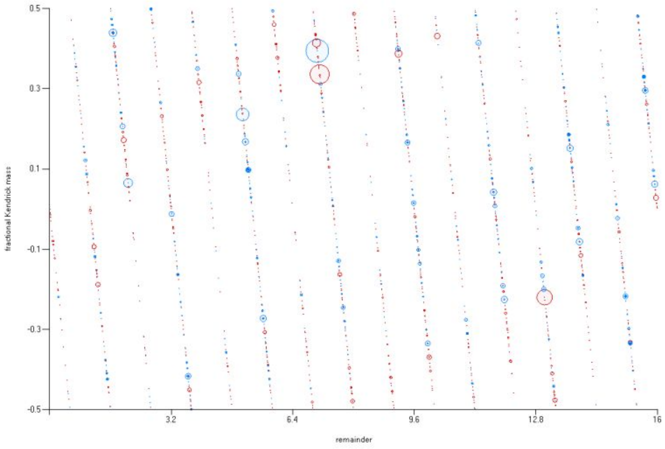

Kendrick plot using “C13H10O” (elemental composition of benzophenone) as the rescaling unit and x=182

“PBRxn_NoUVBP_NaAu”

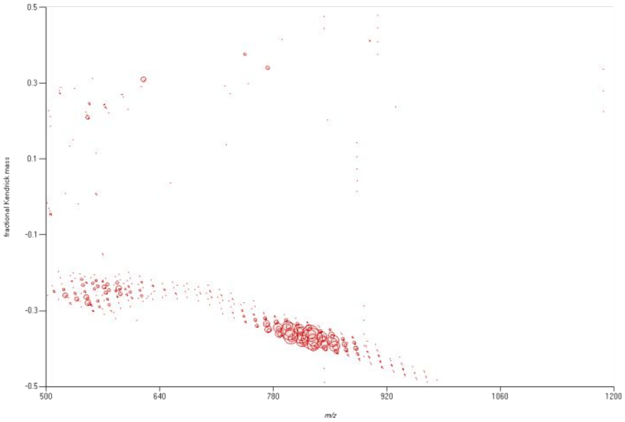

“PBRxn_UVBP_NaAu”

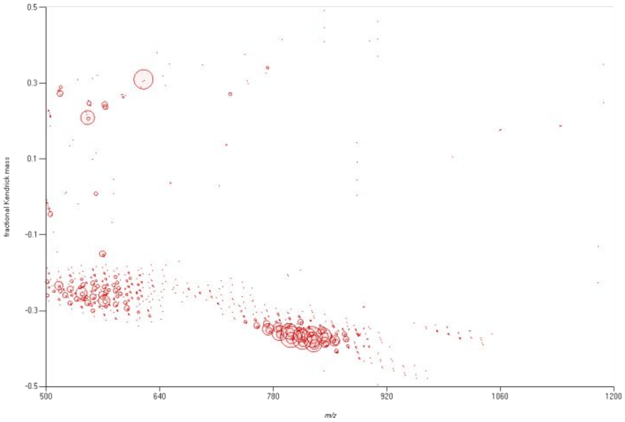

Kendrick plot using “C13H10O” (elemental composition of benzophenone) as the rescaling unit and x=181
The green arrow represents the theoretical variation of m/z and Kendrick mass defect by the addition of one benzophenone moiety

“PBRxn_NoUVBP_NaAu”

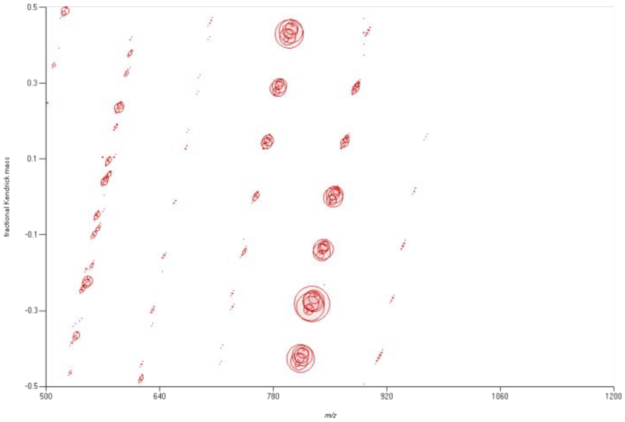

“PBRxn_UVBP_NaAu”

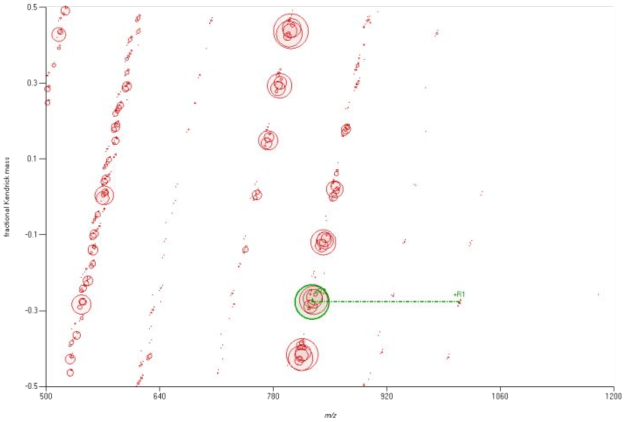

Author's Response to Peer Review Comments:

Thank you so much for all your help! Here we submit the clean format-fixed (we think) version. Let me know if we need anything else.
